# Microenvironmental entropy dynamics analysis reveals novel insights into Notch-Delta-Jagged decision-making mechanism

**DOI:** 10.1016/j.isci.2024.110569

**Published:** 2024-08-21

**Authors:** Aditi Ajith Pujar, Arnab Barua, Partha Sarathi Dey, Divyoj Singh, Ushasi Roy, Mohit Kumar Jolly, Haralampos Hatzikirou

**Affiliations:** 1 Department of Bioengineering, Indian Institute of Science, Bangalore 560012, India; 2 Undergraduate Program, Indian Institute of Science, Bangalore 560012, India; 3 Tata Institute of Fundamental Research, Hyderabad 500046, India; 4 Mathematics Department, Khalifa University, P.O. Box: 127788, Abu Dhabi, UAE; 5 Technische Univesität Dresden, Center for Information Services and High Performance Computing, Nöthnitzer Straße 46, P.O. Box: 01062, Dresden, Germany

**Keywords:** Molecular network, Biocomputational method, Flux data, *In silico* biology, Biological constraints

## Abstract

Notch-Delta-Jagged (NDJ) signaling among neighboring cells contributes crucially to spatiotemporal pattern formation and developmental decision-making. Despite numerous detailed mathematical models, their high-dimensionality parametric space limits analytical treatment, especially regarding local microenvironmental fluctuations. Using the low-dimensional dynamics of the recently postulated least microenvironmental uncertainty principle (LEUP) framework, we showcase how the LEUP formalism recapitulates a noisy NDJ spatial patterning. Our LEUP simulations show that local phenotypic entropy increases for lateral inhibition but decreases for lateral induction. This distinction allows us to identify a critical parameter that captures the transition from a Notch-Delta-driven lateral inhibition to a Notch-Jagged-driven lateral induction phenomenon and suggests random phenotypic patterning in the case of lack of dominance of either Notch-Delta or Notch-Jagged signaling. Our results enable an analytical treatment to map the high-dimensional dynamics of NDJ signaling on tissue-level patterning and can possibly be generalized to decode operating principles of collective cellular decision-making.

## Highlights

• LEUP-based low-dimensional modeling recapitulates NDJ spatiotemporal patterns
• Abstraction of the NDJ pathway into a cell decision-making process
• Identifying a key parameter for transitioning from lateral inhibition to induction
• Analytical results map mechanistic pathway models to the LEUP framework

## Graphical abstract

**Figure:**
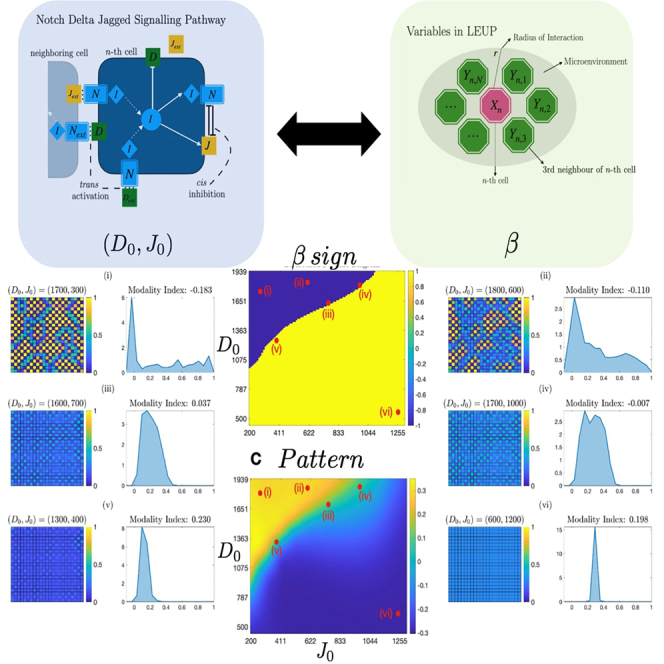

## Introduction

The Notch signaling pathway is evolutionarily highly conserved across species and is crucial during many steps of embryonic development as well as in maintaining tissue homeostasis during adulthood.^1^ It is a cell-cell communication pathway and contributes to cellular proliferation, differentiation, and apoptosis.^2^^,^^3^ Thus, its dysregulation leads to various developmental defects and can also be critical in cancer progression.^1^ In cancer, it can promote metastasis and the associated phenomenon of epithelial-mesenchymal transition (EMT).^1^^,^^4^

The Notch signaling pathway is juxtacrine, i.e., the extracellular domain of the Notch receptor of one cell binds to Delta and/or Jagged family ligands of the neighboring cells (trans-activation). This binding enables the proteolytic cleavage of Notch, whose intracellular domain—Notch intracellular domain (NICD)—then trans-locates to the nucleus to initiate downstream signaling. Notch-Delta signaling between two neighboring cells leads to lateral inhibition, where two cells adopt opposite fates—one acts as a sender (high Delta, low Notch) and the other as receiver (high Notch, low Delta).^5^ However, Notch-Jagged signaling between two cells often leads to lateral induction, where both cells acquire a similar phenotype of being a hybrid sender/receiver (high Notch, high Jagged). Thus, the spatiotemporal dynamics of Notch-Delta-Jagged signaling contributes to various instances of tissue-level pattern formation and has attracted significant attention in computational modeling.^6^^,^^7^^,^^8^

Most computational models developed for Notch signaling so far have incorporated multiple parameters and are high dimensional in nature in terms of variables (Delta, Notch, Jagged, NICD, glycosyltransferase Fringe, NICD targets etc.) considered,^8^^,^^9^^,^^10^^,^^11^^,^^12^^,^^13^^,^^14^ thus making it difficult for any analytical treatment of the same. Thus, identifying the fundamental design principles of Notch signaling-driven cellular decision-making in a low-dimensional space remains challenging.

Cellular decision-making incorporates various factors: (1) internal components of the cell (metabolite levels, structure of gene regulatory network, etc.), (2) external components (biochemical and biophysical microenvironment of the cell, density of neighboring cells, etc.), and (3) noise or uncertainty in sensing the cellular microenvironment, and in various biochemical processes happening inside the cell.^15^ Although single-cell decision-making has been studied meticulously, the study of cellular decision-making on a multi-cellular level remains obscure. Although from a different approach, the intracellular and inter-cellular communications have been understood using the *Hopefield network*.^16^^,^^17^^,^^18^ To address this, we recently proposed a principle known as the least micro-environmental uncertainty principle (LEUP).^19^ The principle is based on the statistical mechanical premise, which can be applied in different biological contexts: collective cell migration, cell differentiation, cellular sensing dynamics, and go-or-grow dynamics.^20^^,^^21^^,^^22^^,^^23^ It adopts the idea of Bayesian inference to construct a mathematical framework. Although there are high-throughput technologies that give us static pictures of a cell’s internal variables, the knowledge of the dynamics of those variables is partial.^23^ LEUP attempts to compensate for this missing knowledge by employing a variational principle on the probability distribution of a cell’s microenvironment which arises from two factors: (1) a cell’s uncertainty in sensing its microenvironment and (2) the phenotypic distribution in the cell population. This is codified in a microenvironmental entropy term. LEUP asserts that the variational principle leads to an ordinary differential equation (ODE) description of the dynamics of the internal cellular state in terms of microenvironmental entropy. The inspiration behind this principle comes from Friston’s free energy principle^24^ and Bayesian brain hypothesis.^25^ Analogous concepts have also been proposed in the seminal works of William Bialek.^26^

In this work, we introduce a sensitivity parameter that quantifies a cell’s capacity for sensing its environment (through ligand binding, mechano-sensing, pseudopodia extension, ion channels, etc.). We derive the relationship between this sensitivity parameter and the dynamics of the cell’s internal and external variables. By demonstrating how cellular behavior depends on the ratio of the biophysical forces and variance of the microenvironmental probability distribution, we predict the phenotypic distribution of a population of cells from varying degrees of information about cell-cell interaction and cell-autonomous variables.

In the context of the Notch signaling pathway, we show that the tissue-level patterning due to Delta-driven lateral inhibition and Jagged-driven lateral induction can be described in terms of the time evolution of local entropy in different regimes of the sensitivity parameter. Specifically, in lateral inhibition (induction), the sensitivity parameter has a negative (positive) value and the local entropy increases (decreases) over time. In scenarios where neither Notch-Delta nor Notch-Jagged signaling dominates, the parameter approaches zero, resulting in the emergence of random patterns.

Hence, we have been able to map the high-dimensional mathematical model to a low-dimensional description amenable to analytical treatment. Our formalism can be generalized for elucidating the operating principles governing collective cellular decision-making processes. Additionally, it holds promise for providing novel insights into specific biological phenomena, as exemplified in this study.

This article has been arranged in the following way: in STAR Methods, we review the Bayesian decision-making and the derivation of the phenotypic Langevin equation. Furthermore, we calculate the sensing intensity parameter using a small-noise approximation applied to the Notch-Delta-Jagged model. After that, in Section results we study the pattern formation and phase transition regimes for different values of sensitivity β and interaction radius r in square lattices and later compare with the mechanistic model of the Notch-Delta-Jagged system. Finally, we discuss and conclude the outcomes of our analyses in Section discussion.

## Results

**Figure 1:**
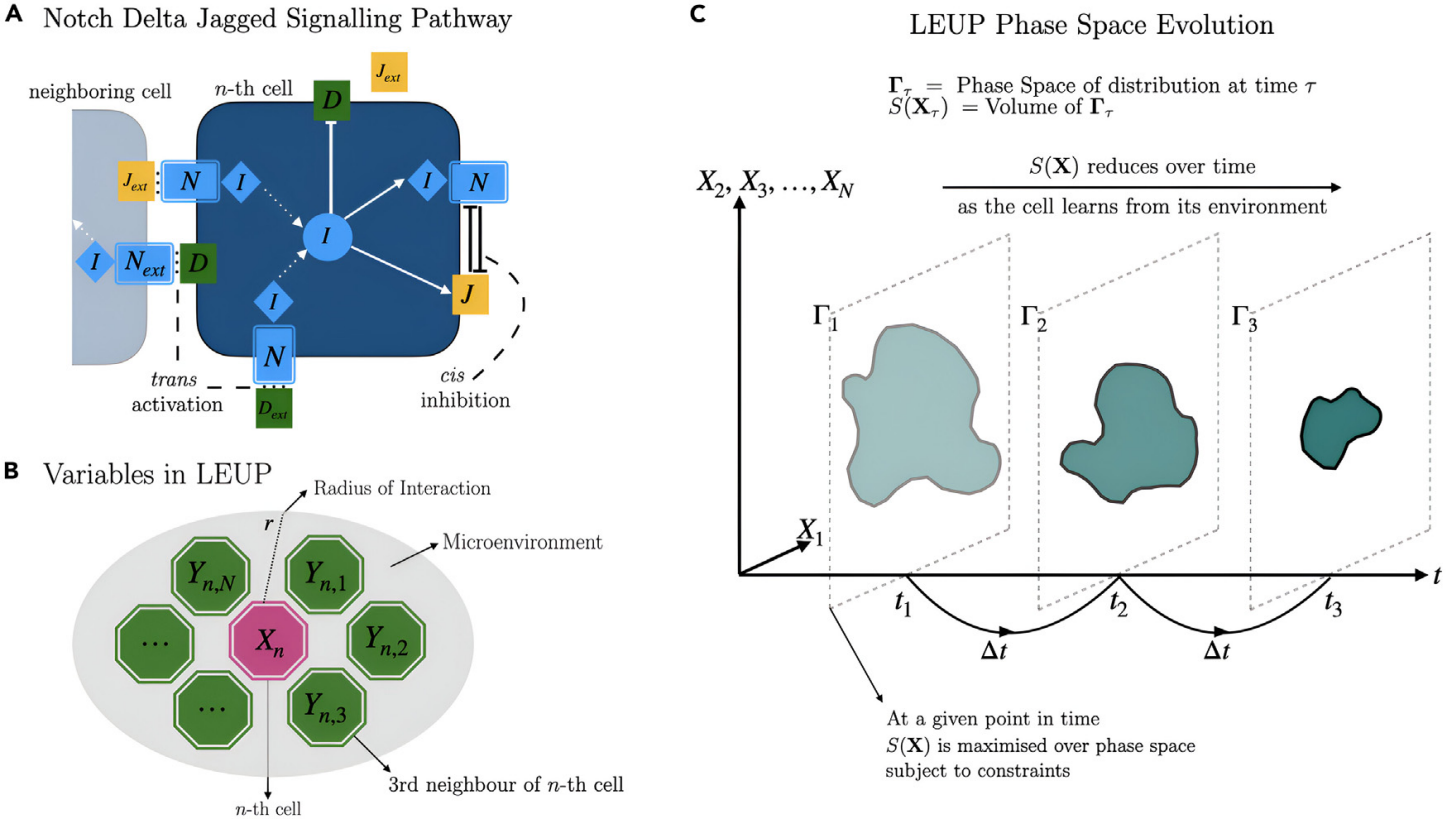
Schematic diagrams representing the two systems studied here (A) This illustrates the Notch-Delta-Jagged signaling pathway. The trans-membrane protein Notch (N) can undergo trans-activation with ligands Delta (D) and Jagged (J) releasing NICD (I), a protein marker that then inhibits D expression and activates N and J production. When both components of this receptor protein-ligand complex are from the same cell, they simply degrade without NICD release, i.e., they undergo *cis*-inhibition. This schematic is inspired by Boareto et al.^27^ (B) This represents the LEUP-based phenomenological model which has only two parameters—a radius of interaction r and sensitivity β. Each cell senses its internal state (phenotype), $X_{n}$, and external variables ($Y_{n}$’s) which are the phenotypes of its neighboring cells. (C) The principles governing LEUP are illustrated. S(X), the entropy of the cell population’s phenotype, reduces over time, which can be visualized as phase space volume over $X_{1},\ldots ,X_{N}$ decreasing over time.

At first, we shall summarize the local microenvironmental information for the Notch-Delta-Jagged system, and then we shall compare the behavior of the LEUP-driven Langevin equation on the lattice. The schematics have been described in Figure 1.

### Sensitivity β and interaction radius r induce phase transitions with respect to microenvironmental entropy

The first question that we attempt to answer is how sensitivity parameter β and interaction radius r impact the dynamics of microenvironmental entropy and pattern formation in LEUP spatiotemporal simulations. Despite the low dimensionality of the parametric space, only r and β, the LEUP systems display a wealth of behaviors.

**Figure 2:**
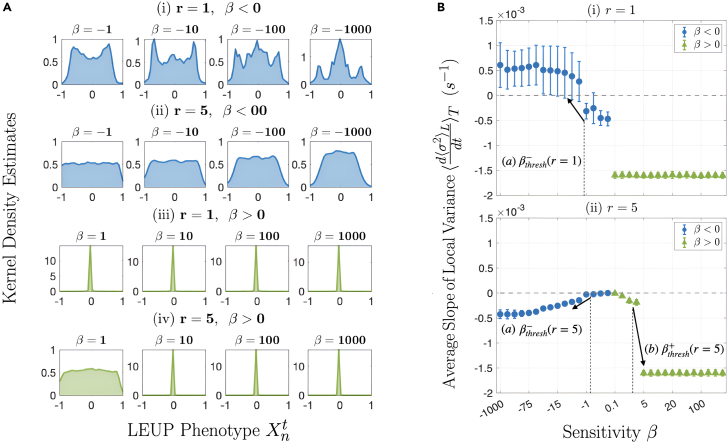
Phenotype distribution and the average rate of change of microenvironment uncertainty over time are examined (A) Kernel density estimates (histograms smoothened with Gaussian kernels) are plotted for different LEUP cell populations. The x axis is the range of phenotypes, $X_{n}^{t}\in \left[-1,1\right]$, and the y axis is the phenotype distribution, mostly normalized to be in [0,1], exceeding 1 in cases where the peaks are too sharp (β>0 populations). As |β| increases (i) (r=1,β<0) populations go from bimodal to trimodal, showing increased sensitivity selects for the hybrid $X_{n}^{t}=0$ state. In (ii) and (iv), the existence of a threshold sensitivity $\left|\beta_{thresh}\right|$ is clearly delineated, only beyond which cell populations are unimodal/very sharply peaked instead of uniformly randomly distributed. (B) These plot the average slope of ${\left\langle \sigma^{2}\right\rangle }_{L}$ over time, i.e., ${\left\langle \frac{d{\left\langle \sigma^{2}\right\rangle }_{L}}{dt}\right\rangle }_{T}$ averaged over time, for each β for different r. For (i) r=1, $\beta_{thresh}^{-}$ is observed and marked by (a). It is the sensitivity beyond which a noticeable increase in ${\left\langle \frac{d{\left\langle \sigma^{2}\right\rangle }_{L}}{dt}\right\rangle }_{T}$ shows the cells beginning to sense. (ii) r=5. Here, both $\beta_{thresh}^{-}$ and $\beta_{thresh}^{+}$ are marked by a decrease in ${\left\langle \frac{d{\left\langle \sigma^{2}\right\rangle }_{L}}{dt}\right\rangle }_{T}$, denoted by (a) and (b). We can see that the transition to informed decision-making is more sharp for β>0, evidenced by the sharp drop in ${\left\langle \frac{d{\left\langle \sigma^{2}\right\rangle }_{L}}{dt}\right\rangle }_{T}$. Throughout this figure, blue (green) plots correspond to negative (positive) β.

First, we investigate the impact of the parameters on the steady-state distribution of the phenotypic parameter $X_{n}^{t}$. We obtain these distributions by constructing the histogram of phenotypes over all lattice nodes. For negative sensitivity β and nearest neighborhood interactions r=1, we observe the emergence of multimodal distributions. In particular, for decreasing β<0 a transition from bimodality to trimodality occurs. Interestingly, this effect is lost for higher radii such as r=5. In the β>0 case, we observe a highly concentrated distribution around the $X_{n}^{t}=0$ value. This implies that the majority of lattice nodes acquire a “hybrid” phenotype. These results can be readily seen in Figure 2A.

Now, we focus on the impact of β on the local variance growth rate, which is a proxy of a temporal average of microenvironmental entropy rate, for difference sensing radii (Figure 2B). When cells sense the immediate neighborhood r=1, for negative sensitivity we observe positive growth rates of the observable ${\left\langle \frac{d{\left\langle \sigma^{2}\right\rangle }_{L}}{dt}\right\rangle }_{T}$. This is consistent with the multi-stable steady-state distributions. On the contrary, for β>0 the local variance decays in time implying that the steady state approximates a delta distribution. The latter is consistent for larger radii for instance for r=5. However, for large sensing radii the ${\left\langle \frac{d{\left\langle \sigma^{2}\right\rangle }_{L}}{dt}\right\rangle }_{T}$ stays non-positive for variations of β. Also, the behavior is more complex exhibiting a non-monotonic behavior.

**Figure 3:**
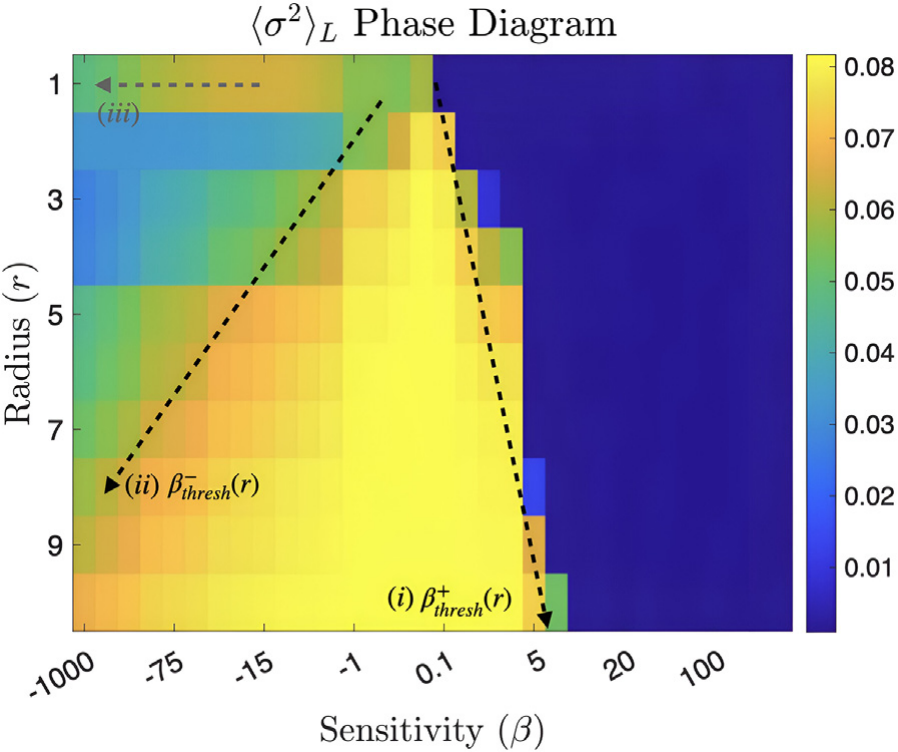
The phase space diagram of the average microenvironmnental uncertainty as a function of sensing radius and sensitivity The steady-state ${\left\langle \sigma^{2}\right\rangle }_{L}$ values are plotted across the phase space of the LEUP model. The increase in $\left|\beta_{thresh}\right|$ as r increases is very evident, marked by (i) for β>0 and (ii) for β<0. A third gray arrow (iii) shows the increase (corresponding to the cells beginning to sense for sensitivity beyond $\beta_{thresh}^{-}\left(r=1\right)$) and gradual decrease in ${\left\langle \sigma^{2}\right\rangle }_{L}$ as trimodality emerges.

To further investigate the latter observation, we plot the steady-state local variance ${\left\langle \sigma^{2}\right\rangle }_{L}$ for a large combination of sensitivity parameter β and the radius r in Figure 3 (also see Figure S1). Please note that the local variance of microenvironmental phenotypes has been calculated using the Moore neighborhood over all the lattice nodes and the averages have been taken over the ensembles of different realization close to the steady states only. Details of these simulations can be found in Figure S1. For any radius and for large enough $\beta >\beta_{thresh}^{+}$, we observe always an unimodal delta distribution of minimal entropy. For radii r>1 and sensitivities $\beta_{thresh}^{-}\leq \beta \leq \beta_{thresh}^{+}$, the local variance stays high. However, when β crosses the lower bound $\beta_{thresh}^{-}$, we observe again a microenvironmental variance reduction. Interestingly, for r=1 case, there is simply an abrupt phase transition from high to low ${\left\langle \sigma^{2}\right\rangle }_{L}$ when β crosses threshold $\beta_{thresh}^{+}$.

### Lateral inhibition (induction) leads to increasing (decreasing) microenvironmental entropy dynamics

**Figure 4:**
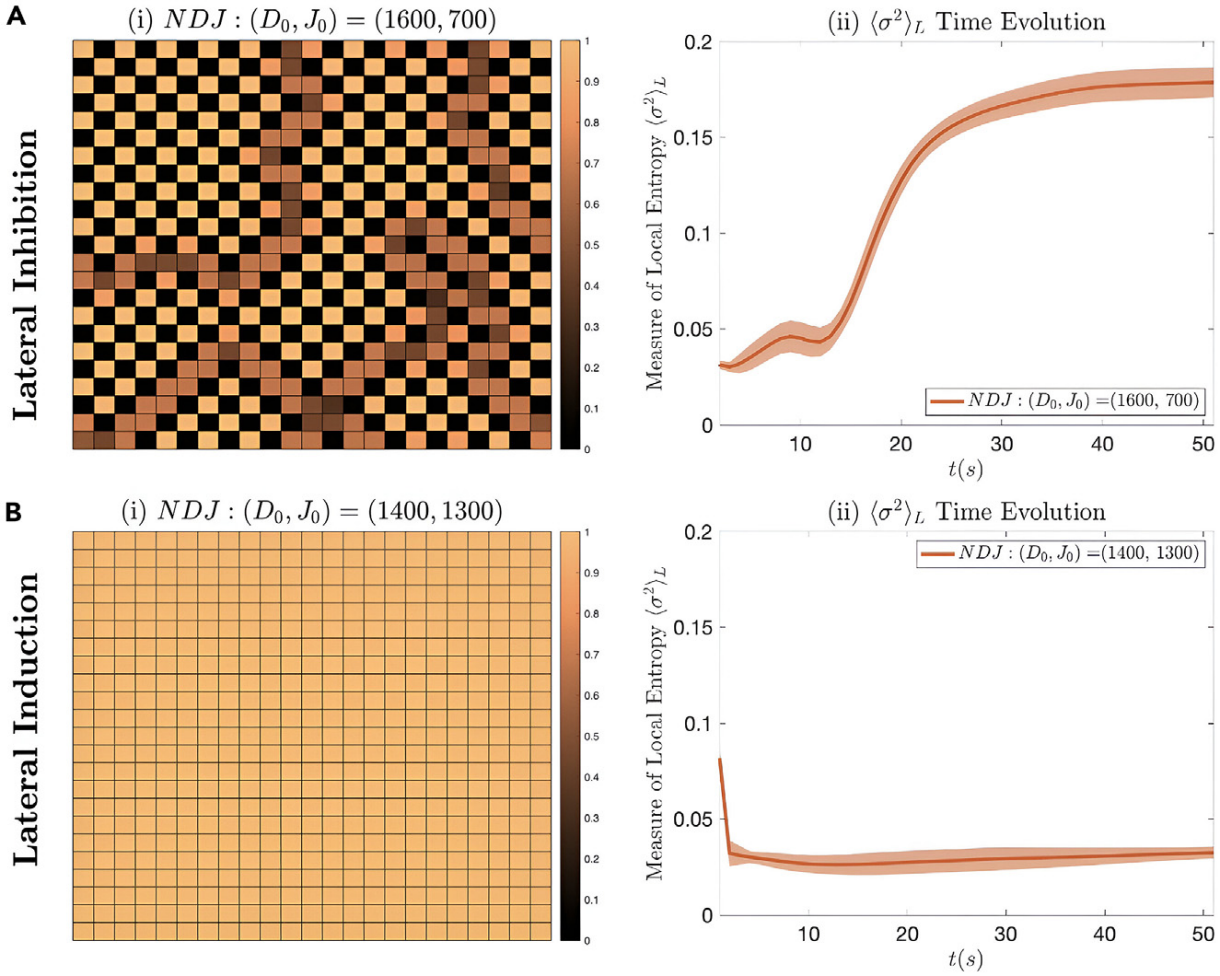
The two Notch-Delta-Jagged signaling regimes are shown (A) Lateral inhibition (checkerboard spatial patterning) and (B) lateral induction (uniform spatial patterning) are observed in (i) Notch-Delta heatmaps of NICD levels. These are normalized to be between [0,1]. Besides patterning, the time evolution of ${\left\langle \sigma^{2}\right\rangle }_{L}$ has also been plotted for both cases. It increases for lateral inhibition (A(ii)) and decreases for lateral induction (B(ii)) over time.

The Notch-Delta-Jagged signaling system has been extensively studied and analyzed.^4^^,^^27^ However, the NDJ system has not been analyzed on the basis of local entropic dynamics. Here, we conduct spatial simulations of Equation 17 to understand the behavior of the microenvironmental entropy, in terms of local variance ${\left\langle \sigma^{2}\right\rangle }_{L}$ (as introduced in the STAR Methods). We consider the NICD levels to correspond to the phenotype of the cells. In turn, we plotted ${\left\langle \sigma^{2}\right\rangle }_{L}$ as a function of time for the Delta-dominated *lateral inhibition* regime (Figure 4A(i)) and the Jagged-dominated *lateral induction* regime (Figure 4B(i)).

The simulations have shown that, for lateral inhibition, ${\left\langle \sigma^{2}\right\rangle }_{L}$ increased over time as shown in Figure 4A(ii). For the lateral induction regime, where Jagged is overexpressed, we observe a decrease in the local variance over time (Figure 4B(ii)). This indicated that the NDJ system agrees with the LEUP premise that microenvironmental should decrease or increase for certain parameter constellations. In the following, we delve deeper into the connection between local entropy and spatial dynamics.

### LEUP recapitulates a noisy NDJ spatial patterning

The Notch-Delta-Jagged mechanism induces distinct patterns when Jagged is overexpressed or not, as seen in Figure 4. In particular, the lateral induction dynamics result into a homogeneous spatial distribution of hybrid phenotype, whereas the lateral inhibition induces the infamous checkerboard-like patterns. The question is if an LEUP model, with only two parameters, is able to capture these two equilibrium patterns.

**Figure 5:**
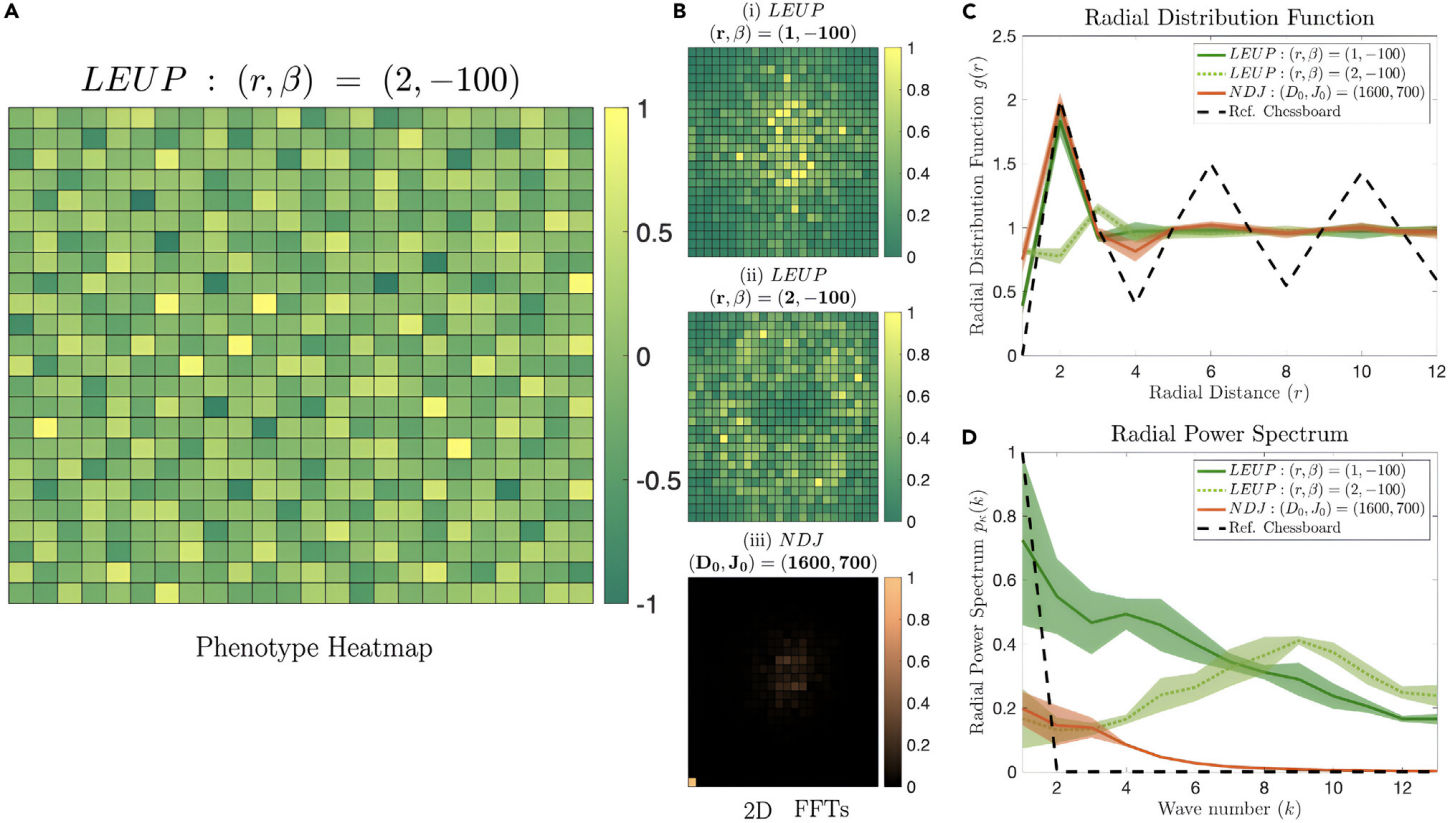
The phenotypic map of the cells is studied in terms of radial distribution function (A) Heatmap of LEUP phenotypes for (r=2,β=−100) shows isolated “islands” of extrema cells $\left(X_{n}^{t}=\pm 1\right)$ surrounded by hybrid cells $\left(X_{n}^{t}=0\right)$. (B–D) (B) Heatmaps of spatial Fourier transforms of (i) chessboard patterned LEUP (r=1,β=−100), (ii) LEUP (r=2,β=−100), and (iii) chessboard-patterned NDJ $\left(D_{0},J_{0}\right)=\left(1600,700\right)$ are plotted. (i) and (iii) correspond to similar spatial patterns and are also alike, with high contributions from a few low frequencies due to spatial ordering. However, the ring-like structure present in (ii) shows that non-trivial spatial order exists in the (r>1,β<0) systems as well. To characterize the spatial patterning, the (C) radial distribution functions and the (D) radial power spectra of the three lattice systems are plotted, along with those of a perfect chessboard pattern for reference. While (i) and (iii) showed reasonably good correspondence with the reference chessboard as expected, the structure in (ii) shows in the power spectra. Whereas the remaining power spectra peaked at low values of r in the frequency domain, (corresponding to low frequencies), LEUP (r=2,β=−100) systems peak at a noticeably larger radius (in frequency domain), corresponding to larger frequencies.

Regarding the lateral induction case, we observe, when $\beta >\beta_{thresh}^{+}$, a uniform distribution of phenotypes $X_{n}=0$ emerges. This corresponds to a uniform pattern exactly like the one of the Jagged-dominated NDJ system. In the case of lateral inhibition, things are not so trivial. The NDJ system typically produces frustrated chessboard patterns, whereas the LEUP simulation produces a noisy version of them. In order to quantitatively compare NDJ and LEUP patterns, we invoke the radial distribution function (rdf) and the spatial fast Fourier transform (FFT). In Figure 5C, we observe that the NDJ is close to the ideal chessboard rdf and the LEUP for r=1 and β<0 is a noisy version of that. The same conclusion can be drawn by observing Figure 5B(i and iii), where the FFT and the corresponding power spectrum show the LEUP and NDJ chessboard-like versions.

Now let us focus on the patterns produced by LEUP when the sensing area has radius r>1. Interestingly, the chessboard pattern is not any more attainable. However, we are obtaining a sparse activation of nodes. In particular, cells expressing extreme phenotypes are almost always surrounded by cells of hybrid $\left(X_{n}^{t}=0\right)$ phenotypes; rarely, there are more than a couple of $X_{n}^{t}=\pm 1$ cells as immediate neighbors. The FFT and the rdf indicate that phenotype activation takes place further away from the immediate neighborhood (Figures 5B(ii), 5C, and 5D). These patterns are called “checkerboard” in the literature. Interestingly, when the NDJ system communicates in larger radii, then the patterns are similar to those observed in developing pancreas patterning, in which the Notch-Delta signaling adopts a *lateral stabilization*^28^ mechanism.

### The sensitivity parameter (β) quantifies the relative impact of intrinsic and extrinsic variables on cell decisions

The sensitivity β quantifies a cell’s ability to sense and integrate information about its microenvironment. Now, we focus on the question of calculating β when the sensing biophysical processes are known and well described by a set of differential equations. To answer this question, for the n− th cell, we consider the external variables $Y_{n}^{s}$, such as ligand densities, and internal variables, $X_{n}^{s}$, such as receptors or internalized proteins. Let us assume a dynamical system of internal and external variables as

(Equation 1) $$\begin{array}{l} {\dot{X}}_{n}^{t}=G\left(X_{n}^{t},Y_{n}^{t}\right)+\xi_{n}\text{,}\\ {\dot{Y}}_{n}^{t}=F\left(X_{n}^{t},Y_{n}^{t}\right)+\eta_{n}\text{,} \end{array}$$

where the intrinsic flow can be rewritten as $G\left(X_{n}^{t},Y_{n}^{t}\right)=\tilde{G}\left(X_{n}^{t},Y_{n}^{t}\right)-X_{n}^{t}$ and the extrinsic one as $F\left(X_{n}^{t},Y_{n}^{t}\right)=\tilde{F}\left(X_{n}^{t},Y_{n}^{t}\right)-X_{n}^{t}$. To simplify the calculations, we assume the steady-state responses of internal and external variables as

(Equation 2) $$\begin{array}{l} X_{n}^{s}=\tilde{G}\left(X_{n}^{s},Y_{n}^{s}\right)+\xi_{n}\text{,}\\ Y_{n}^{s}=\tilde{F}\left(X_{n}^{s},Y_{n}^{s}\right)+\eta_{n}\text{,} \end{array}$$

where the $X_{n}^{s}$ and $Y_{n}^{s}$ are the corresponding steady states of intrinsic and extrinsic variables, respectively. Here, we assume that the aforementioned noises follow the following multivariate Gaussian distributions $\xi_{n}∼N\left(0,\Sigma_{X_{n}^{s}}\right)$ and $\eta_{n}∼N\left(0,\Sigma_{Y_{n}^{s}}\right)$. The probability distribution of internal variables according to the aforementioned dynamics reads as

(Equation 3) $$P\left(X_{n}^{s}|Y_{n}^{s}\right)=\frac{e^{-\frac{1}{2}\left(X_{n}^{s}-\tilde{G}\left(X_{n}^{s},Y_{n}^{s}\right)\right)\Sigma_{X_{n}^{s}}^{-1}{\left(X_{n}^{s}-\tilde{G}\left(X_{n}^{s},Y_{n}^{s}\right)\right)}^{T}}}{{\left(2\pi \right)}^{\frac{d}{2}}|\Sigma_{X_{n}^{s}}{|}^{\frac{1}{2}}}\text{.}$$

In the small-noise approximation, using similar arguments with Bialek,^26^ we can calculate the connection between the internal and external variable probability distributions (akin to a change of variables during integration).

(Equation 4) $$P\left(X_{n}^{s}\right)\left|\left|\frac{dX_{n}^{s}}{dY_{n}^{s}}\right|\right|=P\left(Y_{n}^{s}\right)\text{.}$$

The quantity $\left|\left|\frac{dX_{n}^{s}}{dY_{n}^{s}}\right|\right|$ corresponds to the absolute value of the determinant of the Jacobian $\left|\nabla_{Y_{n}^{s}}G\left(X_{n}^{s},Y_{n}^{s}\right)\right|$ estimated at the steady state (please note that $\nabla_{Y_{n}^{s}}G\left(X_{n}^{s},Y_{n}^{s}\right)=\nabla_{Y_{n}^{s}}\tilde{G}\left(X_{n}^{s},Y_{n}^{s}\right)$). We substitute the value of Jacobian in the small-noise approximation limit and the value of $P\left(Y_{n}^{s}\right)$ into Equation 4:

(Equation 5) $$P\left(X_{n}^{s}\right)\left|\nabla_{Y}G\left(X_{n}^{s},Y_{n}^{s}\right)\right|=\frac{e^{-\frac{1}{2}\left(Y_{n}^{s}-\tilde{F}\left(X_{n}^{s},Y_{n}^{s}\right)\right)\Sigma_{Y_{n}^{s}}^{-1}{\left(Y_{n}^{s}-\tilde{F}\left(X_{n}^{s},Y_{n}^{s}\right)\right)}^{T}}}{{\left(2\pi \right)}^{\frac{d}{2}}|\Sigma_{Y_{n}^{s}}{|}^{\frac{1}{2}}}$$

The quadratic term related to the external dynamics Gaussian goes to zero because we expect that the random variable $Y_{n}^{s}$ is very close to its expected value $\tilde{F}\left(X_{n}^{s},Y_{n}^{s}\right)$ at steady state. At this point, we use the LEUP steady state as calculated in Equation 12. Entropy of the microenvironment, a d-dimensional multivariate normal distribution, is $\frac{1}{2}\ln\left({\left(2\pi e\right)}^{d}|\Sigma_{Y_{n}^{s}|X_{n}^{s}}|\right)$. Thus,

(Equation 6) $$P\left(X_{n}^{s}\right)=\frac{e^{-\frac{\beta }{2}\ln\left({\left(2\pi e\right)}^{d}|\Sigma_{Y_{n}^{s}|X_{n}^{s}}|\right)}}{Z}=\frac{{|\Sigma_{Y_{n}^{s}|X_{n}^{s}}|}^{-\beta /2}}{Z^{\prime }}$$

where the normalization constants are $Z=\int \ldots \int e^{-\beta S\left(Y_{n}^{s}|X_{n}^{s}\right)}dX_{n}^{s}$ and $Z^{\prime }=\int \ldots \int {|\Sigma_{Y_{n}^{s}|X_{n}^{s}}|}^{-\beta /2}dX_{n}^{s}$. Substituting into Equation 5, we get

(Equation 7) $$\frac{{\left|\Sigma_{Y_{n}^{s}|X_{n}^{s}}\right|}^{-\beta /2}}{Z^{\prime }}=\frac{\left|\nabla_{Y}G\left(X_{n}^{s},Y_{n}^{s}\right)\right|}{{\left(2\pi \right)}^{\frac{d}{2}}|\Sigma_{Y_{n}^{s}}{|}^{\frac{1}{2}}}$$

Taking the logarithm and simplifying gives us

(Equation 8) $$-\frac{\beta }{2}\ln\left|\Sigma_{Y_{n}^{s}|X_{n}^{s}}\right|=-\ln\left|\nabla_{Y}G\left(X_{n}^{s},Y_{n}^{s}\right)\right|-\ln\left(\frac{{\left(2\pi \right)}^{d/2}{|\Sigma_{Y_{n}^{s}}|}^{1/2}}{Z^{\prime }}\right)$$

Here, we estimate the normalization term as $Z^{\prime }={\left|{\Sigma_{Y}}_{n}^{s}\right|}^{-\beta /2}M$, where $M=\int dX_{n}^{s}$ is the capacity concentration of intrinsic variables. Also, we use the definition of entropy to obtain $\ln\left({\left(2\pi \right)}^{d/2}{\left|{\Sigma_{Y}}_{n}^{s}\right|}^{1/2}\right)=S\left(Y_{n}^{s}\right)-d/2$. We further simplify this equation (see sensitivity parameter β calculation for details), and we can finally arrive to write *sensitivity* or β as

(Equation 9) $$\beta =-\frac{\ln\left|\nabla_{Y}G\left(X_{n}^{s},Y_{n}^{s}\right)\right|+S\left(Y_{n}^{s}\right)-c}{I\left(X_{n}^{s}:Y_{n}^{s}\right)}$$

where c=lnM+d/2 is a constant scalar and the mutual information for multivariate Gaussian reads $I\left(X_{n}^{s}:Y_{n}^{s}\right)=\frac{1}{2}\ln\left(\frac{\left|{\Sigma_{Y}}_{n}^{s}\right|}{\left|\Sigma_{Y_{n}^{s}|X_{n}^{s}}\right|}\right)$.

### Sensitivity β encodes transitions from uni- to bimodality in Notch-Delta-Jagged phenotypic distributions

In the previous section, we have derived an analytical relation for the sensitivity parameter β. In particular, it informs us about the phenotypic responses on microenvironmental variations scaled by the degree of intrinsic-extrinsic correlations. Here, we investigate the relevance of β in the context of NDJ phenotypic dynamics.

As we have seen before, the sensitivity parameter controls the transitions from unimodality to multimodality of phenotypic probability distribution function in LEUP simulations. In particular, when β<0, the phenotypic distribution is bimodal or else unimodal. In turn, we simulate the spatial Notch-Delta-Jagged system for varying $\left(D_{0},J_{0}\right)$ while keeping all other parameters constant. These parameters represent the basis production rates of Delta and Jagged ligands and are the primary drivers of either lateral inhibition (Delta-dominated) or lateral induction (Jagged-dominated) cell signaling (as shown by Boareto et al.^4^). We ran simulations for plausible ranges of these production rate, i.e., $\left(D_{0},J_{0}\right)\in {\left[500,2000\right]}^{2}$, subsequently calculated β for each simulation, from Equation 8, where details can be found in the STAR Methods section, and calculated the corresponding modality index (as defined in the STAR Methods section).

**Figure 6:**
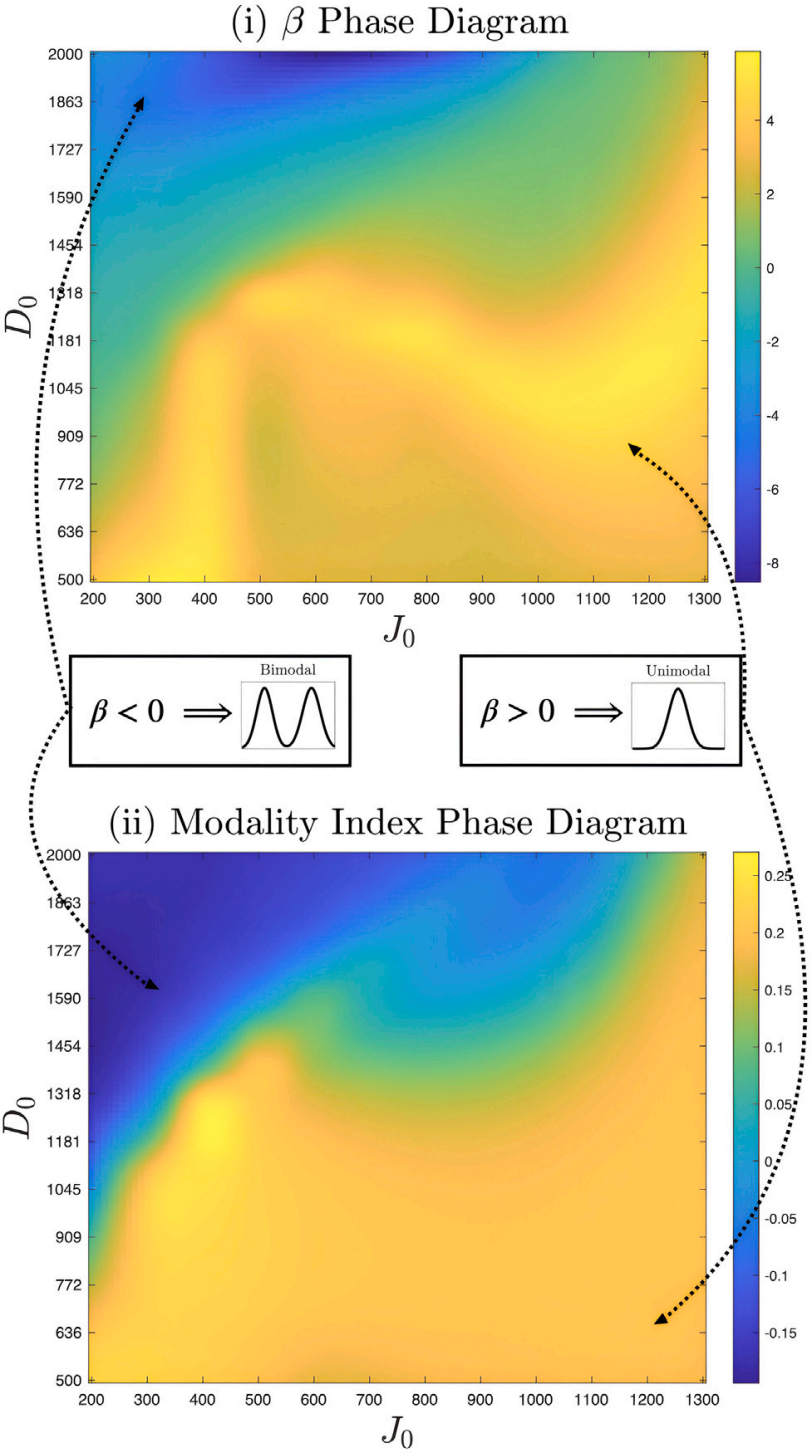
Visualizing sensitivity parameter and modality index in $\left(D_{0},J_{0}\right)$ phase space Here we plot the values of (i) β and (ii) modality index, defined in Equation 22 across the $\left(D_{0},J_{0}\right)$ phase space of the Notch-Delta-Jagged signaling pathway. Negative (positive) values of both order parameters corresponding to bimodal (unimodal) populations are highlighted. While the former is a calculated quantity capturing features of the phase space, the latter is a statistical index that ratifies it.

**Figure 7:**
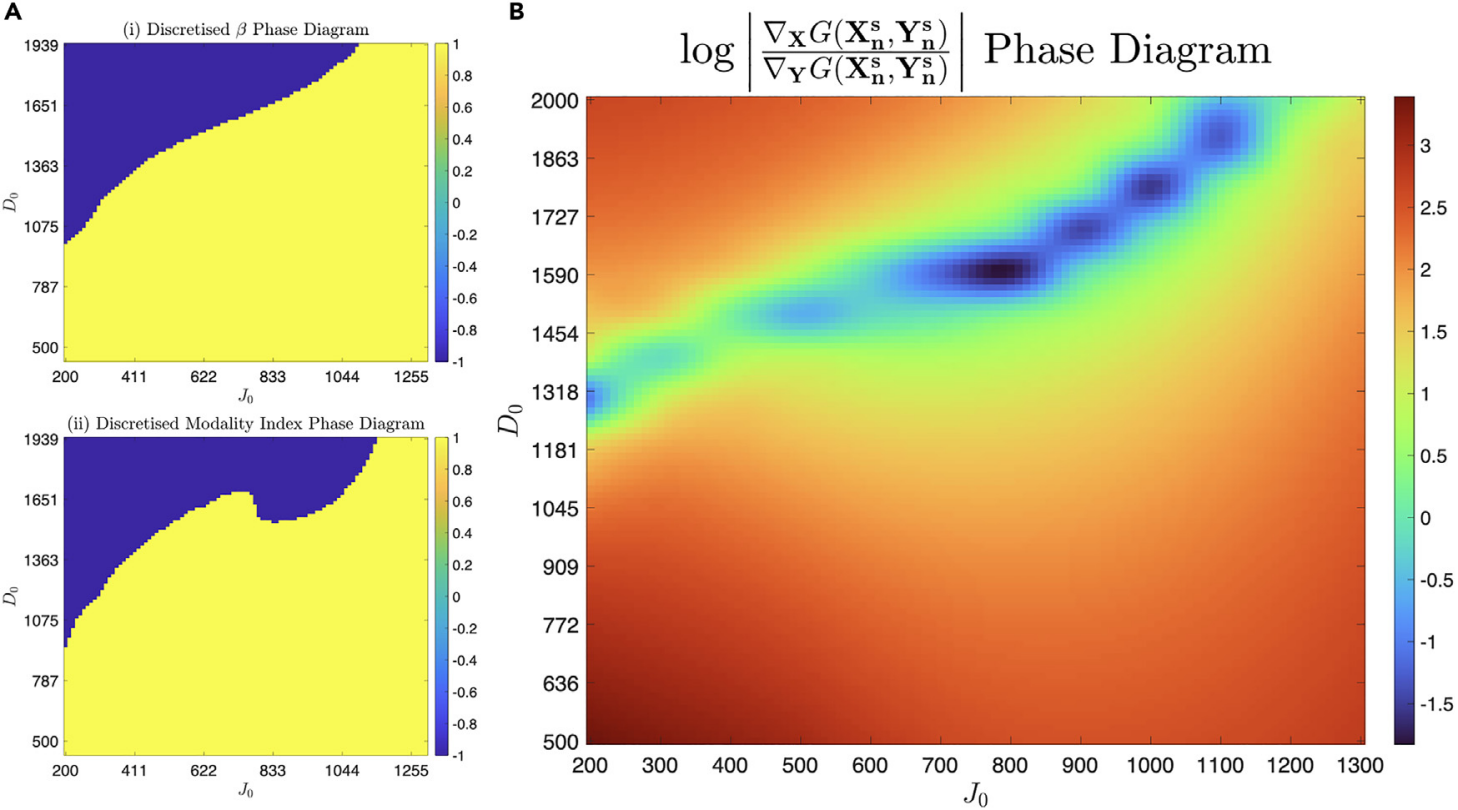
Effect of dynamics of extrinsic and intrinsic variables on phenotypic response (A) The phase diagrams of (i) β and (ii) modality index are discretized by naive thresholding, i.e., all positive (negative) values are equated to +1(−1). This illustrates the phase boundary between the positive and negative regimes. (B) The interpolated phase diagram of a quantity related to the dynamics, $\log\left|\frac{\nabla_{X}G\left(X_{n}^{s},Y_{n}^{s}\right)}{\nabla_{Y}G\left(X_{n}^{s},Y_{n}^{s}\right)}\right|$ is striking—it is only negative along the approximate phase boundary between positive and negative β regimes, corresponding to β=0.

In Figure 6, there is a great correspondence between the phase diagrams of β and the modality index. The simulations that belong to the $\left(D_{0},J_{0}\right)$ bimodality regime correspond to a negative β, and the rest of the phase space indicates unimodality and β<0. The parameter β even captures some non-linear features of the landscape, and the phase space boundary between induction and inhibition bears a remarkably close resemblance between the two. This is more evident in Figure 7A where the boundary of the regimes can be clearly indicated. Finally, we can view β as a quantitative mapping between the LEUP cell decision-making model and the mechanistic NDJ signaling model.

### Combinations of Notch and Jagged production rates lead to random phenotypic decisions (β=0 limit)

In the previous section, we intentionally left out the case of β=0. Interestingly, one can analytically calculate, as shown in the STAR Methods section, that this marginal case is related with the quantity $\ln\left|\frac{\nabla_{X}G\left(X_{n}^{s},Y_{n}^{s}\right)}{\nabla_{Y}G\left(X_{n}^{s},Y_{n}^{s}\right)}\right|<0$. The latter is ratio phenotypic responses when intrinsic over extrinsic variables variations.

Calculating explicitly this aforementioned ratio for different combinations of $\left(D_{0},J_{0}\right)$, we observe that it is negative along the boundary of the two regimes and positive elsewhere (see Figure 7B). This can be interpreted that intrinsic dynamics drive the phenotypic decisions everywhere except the boundary β=0. At the boundary, the cell does not anymore control the decisions/fates and phenotype changes due to microenvironmental fluctuations.

**Figure 8:**
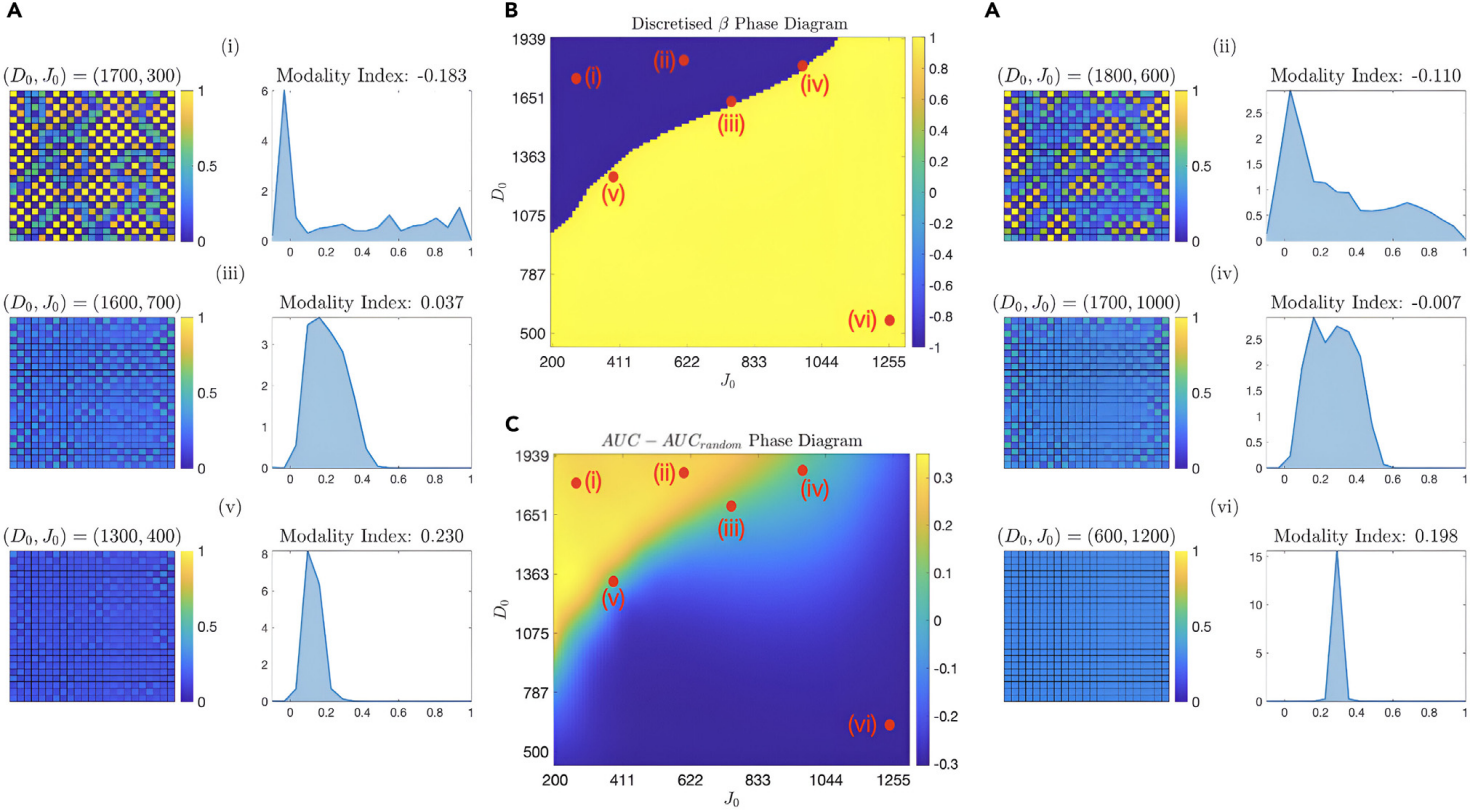
Tissue-level pattern formation The different steady-state configurations of a square lattice of cells correspond to different β values and their population distributions. The color in the heatmaps in the six peripheral plots of (A) represents the level of min-max normalized NICD values $\left(\left(X_{final}-100\right)/\left(1400-100\right)\right)$. The population distributions of NICD values are also captured by kernel density estimates; their modality index (see Equation 22) can be mapped to their β values. (B) is the same as Figure 7A(i), which has a color bar running from −1 to +1. Specifically, the yellow region is β >0 (corresponding to a homogeneous phase of hybrid cells), and the blue region is β <0 (salt-and-pepper islands in a sea of cells of the sender phenotype). For β∼ 0, we see random patterns. This is further underlined by its correspondence with (C), a metric that purely captures the spatial patterning of each population. The metric is the normalized area under the curve of the power spectra of each pattern. The details can be found in Figure S3.

In Figure 8A, we show the different steady-state patterns emerging from different β regimes. We see lateral induction for β >0 cases, resulting in a homogeneous hybrid phase, while, for β <0, we see patches of salt-and-pepper patterned phase surrounded by a sea of cells of the sender phenotype. For β≈ 0, we do not see any discernible pattern. To quantify the deviations of the patterns from a fully random configuration, we calculated the corresponding structure factor, i.e., the power spectrum of the spatial Fourier transform. In turn, we have compared the Area Under the Curve (AUC) between the corresponding pattern of $\left(J_{0},D_{0}\right)$ pair and a purely random pattern (see STAR Methods section). The resulting Figure 8C shows an impressive coincidence with the β sign graph in Figure 8B.

## Discussion

In this article, we proposed a mathematical model of lattice-based phenotypic decision-making. We assumed that each cell in the lattice will change its phenotype according to the entropy of its microenvironment. This model is consistent with the recently formulated LEUP theory for cell decision-making in multi-cellular systems.

We obtained a relation for the sensing intensity parameter that could be interpreted to shed light on its possible origin: in the low noise approximation limit, it (the sensing intensity parameter) is the ratio of logarithmic factors of (1) the gradient of sensing dynamics with respect to microenvironmental variables and (2) the variance of the local microenvironmental distribution. Subsequently, we used the example of Notch-Delta-Jagged signaling to estimate the sensitivity parameters. Using this example, we established the conditions for which it could have three possible cases (i.e., β=0, β<0, and β>0). In the agent-based model of phenotypic decisions, we characterized the pattern formations that appeared due to microenvironmental phenotypic interactions and then compared our model with classical mechanistic Notch-Delta-Jagged signaling. To study this, we use two neighborhoods, i.e., (1) the nearest neighborhood (r=1) and (2) the extended neighborhood (r>1) interactions. For the nearest neighborhood interactions, the distribution of phenotypes changes from bimodal to trimodal distributions; as sensing intensity increases, the frequency of hybrid phenotypes increases. We also found chessboard-like patterns that resemble those found in the mechanistic Notch-Delta-Jagged model. Interestingly, for the extended neighborhood (r>1) interactions, we found unimodal distributions as we escalate the sensing intensity values. Additionally, we used the rdfs and average local variance to quantify spatial patterns in the phenotypes and explored the “fluid”-like structures developed in the phenotypic space. Finally, we have quantitatively established that the sign of the β parameter can encode the patterns produced by NJD simulations (quantified via a function of the corresponding structure factor).

The Notch pathway is ubiquitous in nature and is responsible for various biological processes, viz., embryonic development, wound healing, angiogenesis, and cancer progression.^6^ The intracellular interplay between Notch-Delta-Jagged signaling and transcription factors- and microRNAs-based regulatory network for EMT^4^ during cancer metastasis might result in other different interesting spatiotemporal patterns at the tissue level. This Notch-EMT-combined mechanistic circuit would have a different microenvironment. However, the Notch-related mechanism is not the sole mechanism involved in EMT regulation, since it can be induced also via Wnt/beta pathway (WNT) and transforming growth factor β pathways.^29^ To take into account this regulation ambiguity, we decided to study a generalized mechanism that results in the same phenotypic decision. LEUP offers a generalized theory of cell decision, where EMT could be studied without the exact knowledge of the underlying regulatory mechanisms. This paper is the first step toward this goal since proliferation and motility are not included.

Another interesting aspect of our study regards the finding of the “no decision” boundary. In particular, we found out that there is a family of Delta and Jagged production rates where cell decisions are influenced by microenvironmental fluctuations. This constitutes a testable hypothesis since one could design experiments that modulate accordingly the corresponding production rates and observe the corresponding cell fate decisions. If this is true, it can provide ideas for treating pathologies that depend on impaired Notch-Delta signaling. For example, beyond its crucial role in cancer progression, dysregulation of this pathway has also been implicated in various diseases like pulmonary hypertension and Duchenne muscular dystrophy (for a review, see Siebel et al.^30^). Mutations affecting different components of the pathway have been associated with a spectrum of disorders such as cerebral autosomal dominant arteriopathy with subcortical infarcts and leukoencephalopathy, Alagille syndrome, and spondylocostal dysostosis, among numerous others.^30^

On a technical note, we have assumed that the distribution of the microenvironment follows the Gaussian distribution. However, one can use a different microenvironmental distribution to study patterns emerging from phenotypic decisions. If a strong coupling exists between the microenvironment and the cell, one may use other definitions of entropy like non-ergodic entropy (e.g., Tsallis entropy,^31^ Renyi entropy,^32^ etc.), which will further help us to investigate phenotypic decision-making in a deeper level. Choosing such entropic functionals mandates further theoretical investigations. Finally, another interesting model extension is considering an off-lattice spatial domain to study the effect of LEUP-driven phenotypic decision-making.

An intriguing extension of our research, extending beyond the NDJ pathway, involves mechanisms associated with toggle-switch genes.^33^^,^^34^ These genes are pervasive in developmental processes and also play significant roles in cancer. Toggle-switch mechanisms are characterized by their ability to switch between ON and OFF states, exhibiting bistable behavior similar to that observed in NDJ. Specifically, we calculate our parameter β for various toggle-switch pathways and analyze its relationship to the corresponding phenotypic distribution and pattern formation.

Here, we have not considered the phenomena of cell proliferation or migration. It will be interesting to see how the rate of proliferation can influence the pattern formation regimes. This is particularly interesting for the case of the go-or-grow phenomenon (EMT/Mesenchymal-Epithelial Transition [MET] which have proliferation associated with the epithelial state), which is found in highly invasive cancers such as gliomas.^35^^,^^36^^,^^37^^,^^38^ Moreover, one can study phenotypic decision-making in different topologies (from networks to lattices and spatial geometries). We have used periodic boundary conditions in the simulations. Further work should explore the impacts of other different boundary conditions (e.g., reflective and absorbing boundary conditions, etc.) on phenotypic decision-making on the spatial scale.

In summary, we have shown how microenvironmental uncertainty can drive the phenotypic decision-making on lattice, which further helps us to study phenotypic decision-making when the precise knowledge about the internal states of the cells is unknown and/or partially known.

### Limitations of the study

There exist some limitations of the study, which are as follows. (1) Although the simplification of the entropy-driven principle LEUP is powerful for understanding biological phenomena, it might not fully disclose the multifaceted nature of cellular interactions and signaling pathways. So, one needs biological evidence and knowledge to enhance the theory for further predictions. (2) The exploration of hybrid and trimodal phenotypes is needed in the NDJ model, such that one can encapsulate the complex patterns through LEUP formalism. (3) Lack of experimental pieces of evidence can be an obstacle for this kind of data-driven/information-oriented formalism, which can be tricky in some scenarios.

## Resource availability

### Lead contact

For additional resources or inquiries please kindly contact Prof. Dr. Haralampos Hatzikirou (haralampos.hatzikirou@ku.ac.ae).

### Materials availability

This paper did not include newly generated reagents.

### Data and code availability

• The data used in this paper are publicly available.
• All codes and figures are available publicly on the GitHub page of A.A.P. at https://github.com/aditi-pujar/Microenvironmental-Entropy-Dynamics-in-Notch-Delta-Jagged-Signalling-Context.git.
• For further details needed to re-examine the data analysis and methodology presented in this paper, please kindly request the authors for additional information.

## Acknowledgments

H.H. and A.B. would like to thank 10.13039/501100001663VolkswagenStiftung for its support of the “Life?” program (96732). H.H. has received funding from the 10.13039/501100002347Bundesministerium für Bildung und Forschung under grant agreement no. 031L0237C (MiEDGE project/ERACoSysMed). Finally, H.H. acknowledges the support of the RIG-2023-051 grant from 10.13039/501100004070Khalifa University and the UAE-NIH Collaborative Research grant AJF-NIH-25-KU. M.K.J. was supported by the Ramanujan Fellowship (SB/S2/RJN-049/2018) awarded by the Science and Engineering Research Board, Government of India. U.R. acknowledges the support of the C.V. Raman Fellowship, Indian Institute of Science, Bangalore, and DST-INSPIRE Faculty Fellowship (DST/INSPIRE/04/2022/003052), Government of India.

## Author contributions

Conceptualization, M.K.J. and H.H.; methodology, A.A.P., A.B., P.S.D., D.S., U.R., and H.H.; software, A.A.P., P.S.D., and D.S.; formal analysis A.A.P., A.B., U.R., P.S.D., and D.S.; investigation, A.A.P., A.B., P.S.D., D.S., M.K.J., and H.H.; resources, M.K.J. and H.H.; writing – original draft preparation, all the authors contributed equally; writing – review and editing, all the authors contributed equally; visualization, A.A.P., D.S., and P.S.D.; supervision, M.K.J. and H.H.; project administration, M.K.J. and H.H.; funding acquisition, M.K.J. and H.H. All authors have read and agreed to the published version of the manuscript.

## Declaration of interests

The authors declared no competing interests.

## STAR★Methods

### Key resources table

**Table:** 

REAGENT or RESOURCE	SOURCE	IDENTIFIER
**Software and algorithms**		

Notch-Delta-Jagged signaling mechanistic simulations	This paper	https://github.com/aditi-pujar/Microenvironmental-Entropy-Dynamics-in-Notch-Delta-Jagged-Signalling-Context.git
LEUP theory driven simulations	This paper	https://github.com/aditi-pujar/Microenvironmental-Entropy-Dynamics-in-Notch-Delta-Jagged-Signalling-Context.git
Analytical derivations of LEUP theory	This paper	N/A

### Method details

#### Entropic forces dictate phenotypic dynamics: The LEUP theory

##### Bayesian cell decision-making

We postulate that cellular decision-making is based on two types of information (see Figure 1) the (1) internal variables (i.e. representing genes, RNA molecules, translational proteins, metabolites, receptors, phenotypes, etc.) that contain the local information sensed by a cell and (2) the external variables (i.e. ligands, chemicals, nutrients, cellular density or stress fields, etc.). In a cell population, for the n-th cell at a time t, the external factors are defined as $Y_{n}^{t}\in R^{d\times N}$ , where N is the number of microenvironmental factors, and internal variables of n-th cell at time t are denoted by the vector $X_{n}^{t}\in R^{d}$. The corresponding dimension is the same because the external variables for a cell can be linear combinations of the same internal variables of the neighbouring cell, e.g. RNA expression. We assume the cell as a Bayesian decision maker who tries to estimate the posterior distribution $P\left(X_{n}^{t}|Y_{n}^{t}\right)$ at time t:

(Equation 10) $$P\left(X_{n}^{t}|Y_{n}^{t}\right)=\frac{P\left(Y_{n}^{t}|X_{n}^{t}\right)P\left(X_{n}^{t}\right)}{P\left(Y_{n}^{t}\right)}$$

Here, the local microenvironmental knowledge gained by the cell is represented by the likelihood function $P\left(Y_{n}^{t}|X_{n}^{t}\right)$. This likelihood function is modified by a multiplicative term of the ratio of the probability distribution of internal variables, $P\left(X_{n}^{t}\right)$, the prior and external variables $P\left(Y_{n}^{t}\right)$.

For a cell to construct an accurate likelihood function $P\left(Y_{n}^{t}|X_{n}^{t}\right)$ entails a significant energetic investment by the cell, since its specification requires the production of sufficient membrane receptors. Therefore, defining an informative prior $P\left(X_{n}^{t}\right)$ provides an energetically savvy strategy for cells. A biological example can be the response of the cell in a nutrient-enriched environment. Cells sense their local environment through different biochemical processes, like polymerizing pseudopodia, trans-locating receptor molecules or modifying its cytoskeleton according to mechanical signals.^39^^,^^40^ If cells build an informative prior, then the price for recruitment can be reduced over time t. This precisely the premise of the Least Microenvironmental Uncertainty Principle (LEUP)^20^^,^^21^ which has been linked to the Bayesian Learning in a time independent manner.^23^

##### The Least microEnvironmental Uncertainty Principle (LEUP)

In order to calculate the steady state probability distribution function (pdf) of the internal states of the cell, we developed a maximum entropy principle for cellular internal state distributions which will maximize the internal variable entropy constrained by the mutual information $\bar{I}\left(Y_{n}^{s}:X_{n}^{s}\right)$ between environment and cellular internal variables. The evaluation of the internal states at the steady state in terms of a variational principle can then be written as

(Equation 11) $$\begin{array}{l} \frac{\delta }{\delta P\left(X_{n}^{s}\right)}\{S\left(X_{n}^{s}\right)+\beta [\int P\left(X_{n}^{s}\right)I\left(Y_{n}^{s}:X_{n}^{s}\right)dX_{n}^{s}-\\ \bar{I}\left(Y_{n}^{s}:X_{n}^{s}\right)]-\lambda \left[\int P\left(X_{n}^{s}\right)dX_{n}^{s}-1\right]\}=0\text{,} \end{array}$$

The functional derivative with respect to the internal variables is denoted by $\delta /\delta P\left(X_{n}^{s}\right)$. The constraints associated with the Lagrange multipliers in Equation 11, i.e., β and λ correspond to the target value of the local mutual information $\bar{I}\left(Y_{n}^{s}:X_{n}^{s}\right)$ and the normalization constant of the internal state probability distribution. Extra information regarding internal or external variables from biological experiments can be included in terms of statistical observables as additional constraints. Solving Equation 11, the probability distribution of internal states results in a Gibbs-type distribution:

(Equation 12) $$P\left(X_{n}^{s}\right)=\frac{e^{\beta I\left(Y_{n}^{s}:X_{n}^{s}\right)}}{Z_{I}}=\frac{e^{-\beta S\left(Y_{n}^{s}|X_{n}^{s}=x\right)}}{Z_{s}}\text{,}$$

where the normalization constant is $Z_{I}=\int e^{-\beta I\left(Y_{n}^{s}:X_{n}^{s}\right)}dX_{n}^{s}$ and $Z_{s}$ is defined as $\int e^{-\beta S\left(Y_{n}^{s}|X_{n}^{s}\right)}dX_{n}^{s}$ . In SI 3, we present the details of the calculation. The parameter β encodes the sensitivity of the cell to the microenvironment. From Equation 12, we can see that the quantity $S\left(Y_{n}^{s}|X_{n}^{s}\right)$ is akin to the potential of the system. In the next section, we shall use this analogy to further write the phenotypic Langevin equation for an arbitrary cell.

##### Phenotypic Langevin equation

During cellular decision-making, a cell changes its phenotype (internal state) according to the states of its neighbouring cells (microenvironmental state). Through the Langevin equation, one can describe the phenotypic dynamics of the cell, when interacting with its neighbourhood. We assume that the phenotype of the $n^{th}$ cell at time $t\in R^{+}$ is a continuous variable, i.e., $X_{n}^{t}\in R^{d}$. The external variable, $Y_{n}^{t}\in R^{d\times N}$ is the set of cell phenotypes within a sensing/interaction radius r∈R, i.e. $Y_{n}^{t}=\left(X_{n,1}^{t},X_{n,2}^{t},\ldots ,X_{n,N}^{t}\right)$, where N is the number of neighbors, as shown in Figure 1B.

Moreover, due to the absence of phenotypic inertia, one can consider an overdamped Langevin equation for the phenotypic time evolution. We consider that the rate of change of the phenotype is associated with the gradient of microenvironmental entropy and Gaussian noise (present during the decision-making). So, the noise has a unit variance and zero mean. The properties of the noise are $\left\langle \xi_{n}^{X}\left(t\right)\right\rangle =0$ and $\left\langle \xi_{n}^{X}\left(t_{1}\right)\xi_{m}^{X}\left(t_{2}\right)\right\rangle =2D\delta \left(t_{1}-t_{2}\right)\delta_{mn}$ where $t_{1}$ and $t_{2}$ are the two distinct time points. The diffusion constant in the space of phenotypes is denoted by D. The Delta function is defined by $\delta \left(t_{1}-t_{2}\right)$ and the Kronecker delta by $\delta_{mn}$. Recalling the microenvironmental entropy as being analogous to a potential, the Phenotypic Langevin equation for the n-th cell in a population of cells, with phenotype $X_{n}^{t}$ therefore reads

(Equation 13) $$\frac{dX_{n}^{t}}{dt}=-\beta \nabla_{X}S\left(Y_{n}^{t}|X_{n}^{t}\right)+\xi_{n}^{X}\left(t\right)$$

For a small noise approximation, one can consider the above Equation 13 to then take the form of the following ordinary differential equation:

(Equation 14) $$\frac{dX_{n}^{t}}{dt}=-\beta \nabla_{X}S\left(Y_{n}^{t}|X_{n}^{t}\right)\text{.}$$

Let us assume that each cell senses/samples the corresponding microenvironmental phenotypes from a Gaussian distribution. The noise term in the Langevin’s equation (i.e., Equation 13) is due to the decision-making stochasticity or to the microenvironmental sensing. From here and onwards, we will assume cells as perfect sensors and we will use the zero noise limit case as represented by Equation 14. Assuming that the phenotypes are all scalars (the number of dimensions of the internal variables, d=1), one can write the phenotype of neighboring cells as the external variables for our n-th cell, i.e. $Y_{n,i}^{t}=X_{n,i}^{t},i=1,2,\ldots ,N$ for the N-neighboring cells. Assuming that the microenvironmental entropy term $S\left(Y_{n}^{t}|X_{n}^{t}\right)=\frac{1}{2}\log\left[2\pi e\sigma_{Y_{n}^{t}|X_{n}^{t}}^{2}\right]$, where $\sigma_{Y_{n}^{t}|X_{n}^{t}}^{2}$ is the sample variance of the cell neighborhood, the entropic force simplifies to:

(Equation 15) $$\frac{\partial S\left(Y_{n}^{t}|X_{n}^{t}\right)}{\partial X_{n}^{t}}=\frac{1}{\eta }\left(\frac{X_{n}^{t}}{N}-\frac{1}{N^{2}}\sum_{j}^{N}X_{j}^{t}\right)$$

where,

(Equation 16) $$\eta =\frac{{X_{n}^{t}}^{2}}{N}+\frac{1}{N}\sum_{j}{X_{n}^{t}}^{2}-\frac{2X_{n}^{t}}{N^{2}}\sum_{j}X_{j}^{t}-\frac{1}{N^{2}}{\left(\sum_{j}X_{j}^{t}\right)}^{2}$$

In this mathematical model, the key parameters are (i) the sensing intensity parameter β and (ii) the microenvironmental radius of interaction r. The latter defines the size of the sensing neighborhood, i.e., all the cells within a distance r from a cell will contribute to the RHS of Equation 15 and influence the cell’s decision-making. Variations of the two parameters induce a wealth of collective phenotypic dynamics, as shown in the results section.

#### Mathematical model of Notch-Delta-Jagged signaling

The components of this pathway are the trans-membrane receptor protein, Notch, ligands Jagged and Delta and an intra-cellular factor, NICD (Notch Intra-Cellular Domain). For a given cell, Notch (N) binds to the ligands Delta (D) and Jagged (J) of its neighboring cell. This *trans*-interaction (with other cells) results in the cleavage of the NICD. NICD then goes to the nucleus and indirectly modulates the production of all three proteins. It transcriptionally activates Jagged and Notch and represses Delta. This asymmetric regulation results in different types of patterns in Delta-dominated versus Jagged-dominated systems. $N_{0},D_{0},\text{and}\;J_{0}$ represent the basal production rates of the proteins.

The functions $H^{s+}/H^{s-}$ represent the transcriptional activation/inhibition (They are shifted Hill functions^5^ of the form $H^{s}\left(X\right)=\frac{1+\lambda {\left[X\right]}^{n}}{1+{\left[X\right]}^{n}}$) of the corresponding species due to NICD. The parameter $k_{C}$ corresponds to the *cis*-inhibition rate. Here, the Notch binds to the Delta or Jagged of the same cell and forms a complex and is hence removed from the system. The parameter $k_{T}$ represents the trans-activation rate which corresponds to the interaction with the neighbouring cell’s Notch, Delta, or Jagged. In the last terms, γ represents the basal degradation rate of the species. We assume first-order kinetics for this.

(Equation 17) $$\begin{array}{l} \frac{dN}{dt}=N_{0}H^{s+}\left(I,\lambda_{I,N}\right)-k_{C}N\left(D+J\right)-k_{T}N\left(D_{ext}+J_{ext}\right)-\gamma N\\ \frac{dD}{dt}=D_{0}H^{s-}\left(I,\lambda_{I,D}\right)-k_{C}ND-k_{T}DN_{ext}-\gamma D\\ \frac{dJ}{dt}=J_{0}H^{s+}\left(I,\lambda_{I,J}\right)-k_{C}NJ-k_{T}J_{n}N_{ext}-\gamma J\\ \frac{dI}{dt}=k_{T}N\left(D_{ext}+J_{ext}\right)-\gamma_{I}I \end{array}$$

where $N_{ext}$, $D_{ext}$, $J_{ext}$ refers to the number of Notch, Delta, and Jagged proteins on the parts of the surfaces (that are in contact with the cell) of the neighbouring cells. $\lambda_{I,i}$ (i=N, D, J) denotes the maximum fold changes in the expression of the respective quantities due to NICD.

##### Quantification of microenvironmental entropy/variance in Notch-Delta-Jagged system

Let us formalise the notion of local microenvironmental entropy by defining an order parameter as ${\left\langle \sigma^{2}\right\rangle }_{L}$. We define ${\left\langle \sigma^{2}\right\rangle }_{L}$ to be the average local variance for a population of cells. For our n-th cell with $X_{n}^{t}=X_{n}^{t}$ (the one dimensional phenotype variable) and $Y_{n}^{t}=\left(Y_{n1}^{t},Y_{n2}^{t},\ldots ,Y_{nN}^{t}\right)=\left(X_{n1}^{t},X_{n2}^{t},\ldots ,X_{nN}^{t}\right)$ (external variables are the phenotypes of its N neighbours), the mean external phenotype it senses is $\bar{X_{n}^{t}}$. And their variance is,

(Equation 18) $$\begin{array}{l} \sigma_{n}^{2}=\text{Var}\left(Y_{n}^{t}\right)=\frac{\sum_{i}^{N}{\left(X_{ni}^{t}-\bar{X_{n}^{t}}\right)}^{2}}{N}\text{,}\\ {\left\langle \sigma^{2}\right\rangle }_{L}=\frac{1}{\left|L\right|}\left(\sum_{n\varepsilon L}\sigma_{n}^{2}\right)\text{.} \end{array}$$

where L refers to the set of cell indices on the lattice and the corresponding cardinality |L| is the number of lattice points. Thus, ${\left\langle \sigma^{2}\right\rangle }_{L}$ is the local variance, averaged over the entire lattice.

This is a measure of local entropy. This arose from postulating that in LEUP, the conditional distributions are normally distributed, implying that their entropy is given by a function of the variance.

(Equation 19) $$\begin{array}{cc} S\left(Y_{n}^{t}|X_{n}^{t}=X_{n}^{t}\right) & =\frac{1}{2}\log\left(c\sigma^{2}\left(Y_{n}^{t}|X_{n}^{t}=X_{n}^{t}\right)\right)\\ & =\frac{1}{2}\log\left(c\sigma_{n}^{2}\right) \end{array}$$

(Equation 20) $$\bar{S}\left(Y_{n}^{t}|X_{n}^{t}\right)=\frac{1}{2}\log\left(c{\left\langle \sigma^{2}\right\rangle }_{L}\right)$$

(Equation 21) $$\Rightarrow {\left\langle \sigma^{2}\right\rangle }_{L}={\left(e^{\bar{S}\left(Y_{n}^{t}|X_{n}^{t}\right)}\right)}^{2}/c$$

where $c=2\pi e^{d/2}$. Thus, our average local variance actually is a measure of the constrained conditional entropy, $\bar{S}\left(Y_{n}^{t}|X_{n}^{t}\right)$ at a given point in time.

##### Quantification of distribution multi-modality

The difference between lateral inhibition and lateral induction shows up in the internal state distribution of cell populations; those with Delta-dominant signalling (lateral inhibition) are bimodal whereas those with Jagged-dominant signalling (lateral induction) are unimodal.

Sarle’s Bimodality Index^41^ is a statistical index between 0 and 1 that distinguishes between unimodal, uniformly random and multimodal distributions:

1. $SBC\;>\;0.5\bar{5}$ for multimodal distributions.
2. $SBC=0.5\bar{5}$ for uniformly random distributions.
3. $SBC\;<\;0.5\bar{5}$ for unimodal distributions.

Therefore, we concretely define our *Modality Index* as

(Equation 22) $$\text{Modality}\;\text{Index}=0.5\bar{5}-SBC\text{.}$$

##### Simulation details

Using the Notch-Delta-Jagged mechanistic model and LEUP driven phenotypic Langevin equation, we simulate the ODEs that govern both Notch Delta and LEUP evolution on cells on fixed square lattices (of size 24×24). The time steps taken were dt=0.01s and the simulations were run for 50,000 to 300,000 iterations.

For the LEUP simulations, each cell was assigned a random variable $X_{n}^{t}\in \left[-1,1\right]$ that captured the phenotype of the cell. The LEUP phenotypes then evolved in accordance with our phenotypic Langevin equation, (13) for different parameter regimes (varying interaction radius r and sensitivity β). In the Notch Delta simulations, each cell was initialised with levels of Notch, Delta, Jagged, and NICD for different basal production rates of Delta and Jagged (i.e. $D_{0}$ and $J_{0}$) and evolved over time. While all the proteins were important markers, we chose the NICD level to be the variable that captured the overall state of the cell best. In other words, the Notch Delta Jagged phenotype of a cell was its NICD concentration. Please note that in all simulations we use periodic boundary conditions.

#### Formulating LEUP in terms of constrained mutual information

Recalling the variational Equation 11 and defining,

$$\begin{array}{c} \bar{I}\left(X_{n}^{s},Y_{n}^{s}\right)=S\left(Y_{n}^{s}\right)-\bar{S}\left(Y_{n}^{s}|X_{n}^{s}\right)\\ \Rightarrow \bar{I}\left(X_{n}^{s},Y_{n}^{s}\right)=S\left(Y_{n}^{s}\right)-\bar{S}\left(Y_{n}^{s}|X_{n}^{s}\right) \end{array}$$

The constraint on conditional entropy can be equivalently formulated as a constraint on mutual information. Thus our variational equation becomes,

(Equation 23) $$\frac{\delta }{\delta P\left(X_{n}^{s}\right)}\{S\left(X_{n}^{s}\right)+\beta [\int P\left(X_{n}^{s}\right)\left(S\left(Y_{n}^{s}\right)-S\left(Y_{n}^{s}|X_{n}^{s}\right)\right)dX_{n}^{s}+\left(S\left(Y_{n}^{s}\right)-\bar{S}\left(Y_{n}^{s}|X_{n}^{s}\right)\right)]\}=0$$

Which simplifies to,

$$\frac{\delta }{\delta P\left(X_{n}^{s}\right)}\left[S\left(X_{n}^{s}\right)+\beta \left(\int dX_{n}^{s}P\left(X_{n}^{s}=x\right)I\left(Y_{n}^{s}|X_{n}^{s}=x\right)-\bar{I}\left(Y_{n}^{s}|X_{n}^{s}\right)\right)\right]=0$$

Which implies $P\left(X_{n}^{s}=x\right)=\frac{e^{\beta I\left(Y_{n}^{s}|X_{n}^{s}=x\right)}}{Z_{I}}$. To conclude,

(Equation 24) $$∴P\left(X_{n}^{s}=X_{n}^{t}\right)=\frac{e^{\beta I\left(Y_{n}^{s},X_{n}^{s}=X_{n}^{t}\right)}}{Z_{I}}=\frac{e^{-\beta S\left(Y_{n}^{s}|X_{n}^{s}=X_{n}^{t}\right)}}{Z_{S}}$$

where,

(Equation 25) $$\begin{array}{cc} Z_{I} & =\int e^{+\beta I\left(Y_{n}^{s},X_{n}^{s}=x\right)}dx\\ & =e^{\beta S\left(Y_{n}^{s}\right)}\int e^{-\beta S\left(Y_{n}^{s}|X_{n}^{s}=x\right)}dx\\ & =e^{\beta S\left(Y_{n}^{s}\right)}Z_{S} \end{array}$$

##### Thermodynamic identity

Previously we have derived:

$$\begin{array}{l} P\left(X_{n}^{t}\right)=\frac{e^{\beta I\left(Y_{n}^{s},X_{n}^{s}=x\right)}}{Z_{I}}\\ \Rightarrow \ln\;P\left(X_{n}^{t}\right)=\beta I\left(Y_{n}^{s},X_{n}^{s}=x\right)-\ln\;Z_{I} \end{array}$$

Now,

$$\begin{array}{cc} S\left(X_{n}^{s}\right) & =-\int P\left(X_{n}^{s}=x\right)\ln\;P\left(X_{n}^{s}=x\right)dx\\ & =\ln\;Z_{I}\int P\left(X_{n}^{s}=x\right)dx-\beta \int P\left(X_{n}^{s}=x\right)I\left(Y_{n}^{s},X_{n}^{s}=x\right)dx \end{array}$$

Therefore, we can derive the corresponding thermodynamic identity for the phenotypes s:

(Equation 26) $$S\left(X_{n}^{s}\right)=\ln\;Z_{I}-\beta I\left(Y_{n}^{s},X_{n}^{s}\right)$$

##### Time evolution of ${\left\langle \sigma^{2}\right\rangle }_{L}$

In Figure S1, we provide detailed simulations for the temporal evolution of ${\left\langle \sigma^{2}\right\rangle }_{L}$ for different parametric regimes.

#### Sensitivity parameter β calculation

By definition, we have,

$$\begin{array}{c} Z^{\prime }=\int {\left|\Sigma_{Y_{n}^{s}|X_{n}^{s}}\right|}^{-\beta /2}dX_{n}^{s}\\ ={\left(2\pi e\right)}^{\beta d/2}\int {\left({\left(2\pi e\right)}^{d}\left|\Sigma_{Y_{n}^{s}|X_{n}^{s}}\right|\right)}^{-\beta /2}dX_{n}^{s}\\ ={\left(2\pi e\right)}^{\beta d/2}\int e^{-\beta S\left(Y_{n}^{s}|X_{n}^{s}\right)}dX_{n}^{s} \end{array}$$

From the identity S(X,Y)=S(X)+S(Y|X)≤S(X)+S(Y), we know,

(Equation 27) S(Y|X)≤S(Y)

We now have to deal with positive and negative β separately.

##### The β>0 case

Continuing from Equation 27,

$$\begin{array}{c} \beta S\left(Y_{n}^{s}|X_{n}^{s}\right)\leq \beta S\left(Y_{n}^{s}\right)\\ \Rightarrow \int e^{-\beta S\left(Y_{n}^{s}|X_{n}^{s}\right)}dX_{n}^{s}\geq \int e^{-\beta S\left(Y_{n}^{s}\right)}dX_{n}^{s}\\ Z^{\prime }\geq {\left(2\pi e\right)}^{\beta d/2}e^{-\beta S\left(Y_{n}^{s}\right)}\left(\int dX_{n}^{s}\right) \end{array}$$

Setting $M=\int dX_{n}^{s}$, where M is some characteristic concentration of internal variables, we ultimately arrive at the inequality,

(Equation 28) $$Z^{\prime }\geq Z_{bound}^{\prime }$$

(Equation 29) $$\text{where}\;Z_{bound}^{\prime }={\left|{\Sigma_{Y}}_{n}^{s}\right|}^{-\beta /2}M$$

therefore, for β>0, we obtain a lower bound on its partition function. From Equation 8, we have,

$$\begin{array}{c} \frac{\beta }{2}\left|\Sigma_{Y_{n}^{s}|X_{n}^{s}}\right|=\ln\left|\nabla_{Y}G\left(X_{n}^{s},Y_{n}^{s}\right)\right|+S\left(Y_{n}^{s}\right)-d/2-\ln\;Z^{\prime }\\ \Rightarrow \frac{\beta }{2}\left|\Sigma_{Y_{n}^{s}|X_{n}^{s}}\right|\leq \ln\left|\nabla_{Y}G\left(X_{n}^{s},Y_{n}^{s}\right)\right|+S\left(Y_{n}^{s}\right)-d/2-\ln\;Z_{bound}^{\prime }\\ \Rightarrow \beta \left(\frac{\left|\Sigma_{Y_{n}^{s}|X_{n}^{s}}\right|-\left|{\Sigma_{Y}}_{n}^{s}\right|}{2}\right)\leq \ln\left|\nabla_{Y}G\left(X_{n}^{s},Y_{n}^{s}\right)\right|+S\left(Y_{n}^{s}\right)-c\\ \Rightarrow \beta \left(-I\left(X_{n}^{s},Y_{n}^{s}\right)\right)\leq \ln\left|\nabla_{Y}G\left(X_{n}^{s},Y_{n}^{s}\right)\right|+S\left(Y_{n}^{s}\right)-c\\ ∴\begin{array}{c} \beta \geq -\left(\frac{\ln\left|\nabla_{Y}G\left(X_{n}^{s},Y_{n}^{s}\right)\right|+S\left(Y_{n}^{s}\right)-c}{I\left(X_{n}^{s},Y_{n}^{s}\right)}\right) \end{array} \end{array}$$

We use the property that $I\left(X_{n}^{s},Y_{n}^{s}\right)\geq 0$ in the last step. Thus, the estimate for β we obtain is actually a *lower bound* for β>0.

##### The β<0 case

We can work out the above calculation in an exactly analogous way in order to arrive at,

$$\begin{array}{l} Z^{\prime }\leq Z_{bound}^{\prime }\\ \begin{array}{c} \beta \leq -\left(\frac{\ln\left|\nabla_{Y}G\left(X_{n}^{s},Y_{n}^{s}\right)\right|+S\left(Y_{n}^{s}\right)-c}{I\left(X_{n}^{s},Y_{n}^{s}\right)}\right) \end{array} \end{array}$$

i.e. $Z_{bound}^{\prime }$ is an upper bound for our partition function, and the estimate we derive for β is actually an *upper bound* for β<0.

Thus, by substituting in $Z_{bound}^{\prime }$ for $Z^{\prime }$, we arrive at lower bounds for β>0 and upper bounds for β<0. In other words, we arrive at the lower bounds for |β| in both regimes. In the main text, we are going to use limiting value for all values of β.

##### The β formula for NDJ system

In this section, we shall consider a system of equations representing Notch-Delta-Jagged signaling^5^^,^^27^ with the presence of Gaussian noise. We denote the Notch expression of the n-th cell at time t by $N_{n}^{t}$, its Delta expression by $D_{n}^{t}$, its Jagged expression by $J_{n}^{t}$ and its NICD expression by $I_{n}^{t}$. We have seen how the system evolves in the ODEs outlined in the Mathematical Framework. We define two new variables here, $L_{n}^{t}=D_{n}^{t}+J_{n}^{t}$ and $L_{ext}=D_{ext}+J_{ext}$, the total internal and external ligand concentrations. We obtain how $L_{n}^{t}$ evolves over time by adding the ODEs for $D_{n}^{t}$ and $J_{n}^{t}$. Thus, our new set of variables - Notch $N_{n}^{t}$, Ligand $\left(L_{n}^{t}\right)$ and NICD $\left(I_{n}^{t}\right)$ levels evolve according to the following ODEs -

(Equation 30) $$\begin{array}{l} \frac{dN_{n}^{t}}{dt}=N_{0}H^{s+}\left(I,\lambda_{I,N}\right)-k_{C}N_{n}^{t}L_{n}^{t}-k_{T}N_{n}^{t}L_{ext}-\gamma N_{n}^{t}+\xi_{n}\\ \frac{dL_{n}^{t}}{dt}=D_{0}H^{s-}\left(I,\lambda_{I,D}\right)+J_{0}H^{s+}\left(I,\lambda_{I,J}\right)-k_{C}N_{n}^{t}L_{n}^{t}-k_{T}{L_{n}}^{t}N_{ext}-\gamma L_{n}^{t}+\xi_{n}\\ \frac{dI_{n}^{t}}{dt}=k_{T}N_{n}^{t}L_{ext}-\gamma_{I}I_{n}^{t} \end{array}$$

In the adiabatic approximation, the internal state of the cell changes at a much faster rate than the average of its neighbours. Hence, the external variables are taken as time-independent in Equation 30. The constants $N_{0}$ and $D_{0}$ are the production rates of Notch and Delta proteins, which are measured in the order of molecules/h. $I_{0}$ is the threshold value of the NICD complex Hill function in a number of molecules.^5^^,^^27^

As the timescale of NICD molecule formation happens on a faster time scale one can easily assume that the rate of change of NICD molecules reaches a steady state (faster than Notch, Delta, and Jagged molecules). In the quasi-steady state, the amount of NICD complex molecule can be written as,

(Equation 31) $$I_{n}^{t}=\frac{k_{T}}{\gamma_{I}}{N_{n}}^{t}L_{ext}=\alpha {N_{n}}^{t}L_{ext}$$

The parameter $\alpha =\frac{k_{T}}{\gamma_{I}}$. This approximation holds because NICD degrades rapidly as compared to N, D and J.^42^ Now, we can substitute the value of $I_{n}^{t}$ in Equation 30 to get a simplified equation for Notch-Delta-Jagged signaling as,

(Equation 32) $$\begin{array}{l} \frac{dN_{n}^{t}}{dt}=N_{0}H^{s+}\left(\alpha {N_{n}}^{t}L_{ext},\lambda_{I,N}\right)-k_{C}N_{n}^{t}L_{n}^{t}-k_{T}N_{n}^{t}L_{ext}-\gamma N_{n}^{t}+\xi_{n}\\ \frac{dL_{n}^{t}}{dt}=D_{0}H^{s-}\left(\alpha {N_{n}}^{t}L_{ext},\lambda_{I,D}\right)+J_{0}H^{s+}\left(\alpha {N_{n}}^{t}L_{ext},\lambda_{I,J}\right)-k_{C}N_{n}^{t}L_{n}^{t}-k_{T}{L_{n}}^{t}N_{ext}-\gamma L_{n}^{t}+\xi_{n} \end{array}$$

We expand the first term (the shifted Hill function) in the Taylor series of Equation 32 around the dissociation constant, $I_{0}$. We specifically did the Taylor series expansion around the dissociation constant to understand the switching behaviour of internal states and the correlation between internal and environmental variables. Considering the first two terms in the Taylor series expansion, we can further write Equation 32 approximately as,

(Equation 33) $$\begin{array}{l} \frac{dN_{n}^{t}}{dt}=\frac{\left(\lambda_{I,N}+1\right)N_{0}}{2}+\frac{hN_{0}\left(\lambda_{I,N}-1\right)}{4I_{0}}\left(\alpha N_{n}^{t}L_{ext}-I_{0}\right)-k_{C}N_{n}^{t}L_{n}^{t}-k_{T}N_{n}^{t}L_{ext}-\gamma N_{n}^{t}+\xi_{n}\\ \frac{dL_{n}^{t}}{dt}=\frac{\left(\lambda_{I,D}+1\right)D_{0}}{2}+\frac{hD_{0}\left(\lambda_{I,D}-1\right)}{4I_{0}}\left(\alpha N_{n}^{t}L_{ext}-I_{0}\right)+\frac{\left(\lambda_{I,J}+1\right)J_{0}}{2}+\frac{hJ_{0}\left(\lambda_{I,J}-1\right)}{4I_{0}}\left(\alpha N_{n}^{t}L_{ext}-I_{0}\right)-k_{C}N_{n}^{t}L_{n}^{t}-k_{T}L_{n}^{t}N_{ext}-\gamma L_{n}^{t}+\xi_{n} \end{array}$$

or in the matrix form, the above system of equations can be written as,

(Equation 34) $$\frac{d}{dt}\left(\begin{array}{c} N_{n}^{t}\\ L_{n}^{t} \end{array}\right)=\left(\begin{array}{c} \frac{\left(\lambda_{I,N}+1\right)N_{0}}{2}+\frac{hN_{0}\left(\lambda_{I,N}-1\right)}{4I_{0}}\left(\alpha N_{n}^{t}L_{ext}-I_{0}\right)\\ \ldots -k_{C}N_{n}^{t}L_{n}^{t}-k_{T}N_{n}^{t}L_{ext}\\ \frac{\left(\lambda_{I,D}+1\right)D_{0}}{2}+\frac{hD_{0}\left(\lambda_{I,D}-1\right)}{4I_{0}}\left(\alpha N_{n}^{t}L_{ext}-I_{0}\right)\\ \ldots +\frac{\left(\lambda_{I,J}+1\right)J_{0}}{2}\\ \ldots +\frac{hJ_{0}\left(\lambda_{I,J}-1\right)}{4I_{0}}\left(\alpha N_{n}^{t}L_{ext}-I_{0}\right)-k_{C}N_{n}^{t}L_{n}^{t}-k_{T}L_{n}^{t}N_{ext} \end{array}\right)-\gamma \left(\begin{array}{c} N_{n}^{t}\\ L_{n}^{t} \end{array}\right)+\left(\begin{array}{c} \xi_{n}\\ \xi_{n} \end{array}\right)$$

Absorbing γ into the characteristic time scale of the system, we can now define the internal states as,

(Equation 35) $$X_{n}^{t}=\left(\begin{array}{c} N_{n}^{t}\\ L_{n}^{t} \end{array}\right)\text{,}$$

and external states as,

(Equation 36) $$Y_{n}^{t}=\left(\begin{array}{c} N_{ext}\\ L_{ext} \end{array}\right)$$

Using this definition of internal and external variables, we can write the system of Equation 34 as,

(Equation 37) $$\frac{d}{dt}X_{n}^{t}=G\left(Y_{n}^{t},X_{n}^{t}\right)-X_{n}^{t}+\bar{\xi }$$

where,

(Equation 38) $$\begin{array}{ll} & G=\left(\begin{array}{c} \frac{\left(\lambda_{I,N}+1\right)N_{0}}{2}+\frac{hN_{0}\left(\lambda_{I,N}-1\right)}{4I_{0}}\left(\alpha N_{n}^{t}L_{ext}-I_{0}\right)\\ \ldots -k_{C}N_{n}^{t}L_{n}^{t}-k_{T}N_{n}^{t}L_{ext}\\ \frac{\left(\lambda_{I,D}+1\right)D_{0}}{2}+\frac{hD_{0}\left(\lambda_{I,D}-1\right)}{4I_{0}}\left(\alpha N_{n}^{t}L_{ext}-I_{0}\right)\\ \ldots +\frac{\left(\lambda_{I,J}+1\right)J_{0}}{2}\\ \ldots +\frac{hJ_{0}\left(\lambda_{I,J}-1\right)}{4I_{0}}\left(\alpha N_{n}^{t}L_{ext}-I_{0}\right)-k_{C}N_{n}^{t}L_{n}^{t}-k_{T}L_{n}^{t}N_{ext} \end{array}\right)\\ & =\left(\begin{array}{c} G_{1}\\ G_{2} \end{array}\right)\\ & \bar{\xi }=\left(\begin{array}{c} \xi_{n}\\ \xi_{n} \end{array}\right) \end{array}$$

To find the steady state condition, we take $\frac{dN_{n}^{t}}{dt}=\frac{dL_{n}^{t}}{dt}=0$. Now, we can use the tools previously mentioned in Equation 2 as,

(Equation 39) $$X_{n}^{s}=G\left(Y_{n}^{s},X_{n}^{s}\right)+\bar{\xi }$$

##### Finding the drift term $\nabla_{Y}G\left(X_{n}^{s},Y_{n}^{s}\right)$

In order to calculate β, the dynamics-related term we need to calculate is $\nabla_{Y}G\left(X_{n}^{s},Y_{n}^{s}\right)$, (which takes on the meaning of drift.)

$$\begin{array}{cc} \nabla_{Y}G\left(X_{n}^{s},Y_{n}^{s}\right) & =\left|\begin{array}{cc} \frac{\partial G_{1}}{\partial N_{ext}} & \frac{\partial G_{1}}{\partial L_{ext}}\\ \frac{\partial G_{2}}{\partial N_{ext}} & \frac{\partial G_{2}}{\partial L_{ext}} \end{array}\right|\\ & =\left|\begin{array}{cc} 0 & N_{n}^{s}\left(\frac{hN_{0}}{4I_{0}}\alpha \left(\lambda_{I,N}-1\right)-k_{T}\right)\\ -k_{T}L_{n}^{s} & N_{n}^{s}\frac{hD_{0}}{4I_{0}}\alpha \left(\lambda_{I,D}-1\right)\\ & +N_{n}^{s}\frac{hJ_{0}}{4I_{0}}\alpha \left(\lambda_{I,J}-1\right) \end{array}\right|\\ & =k_{T}^{2}\left(\frac{N_{0}h\left(\lambda_{I,N}-1\right)}{4I_{0}\gamma_{I}}-1\right)\left|N_{n}^{s}L_{n}^{s}\right| \end{array}$$

This finally simplifies to,

(Equation 40) $$\nabla_{Y}G\left(X_{n}^{s},Y_{n}^{s}\right)=\left|k_{T}\kappa \left|N_{n}^{s}L_{n}^{s}\right|\right|$$

where, $\kappa =k_{T}\left(1-\frac{h_{N}N_{0}\left(\lambda_{N}-1\right)}{4I_{0}}\right)$. (This is a convenient way to define these parameters because of a later calculation involving $\nabla_{X}G\left(X_{n}^{s},Y_{n}^{s}\right)$). The values of the parameters we used have been derived from^5^^,^^27^ and have been listed in the S.I.

#### Numerical calculation of β from NDJ simulations

We now proceed to perform the above calculation for a given point in the Notch Delta Jagged phase space. Recalling the (fully expanded) β formula,

$$\beta =-\left(\frac{\ln\left|\nabla_{Y}G\left(X_{n}^{s},Y_{n}^{s}\right)\right|+S\left(Y_{n}^{s}\right)-\left(\ln\;M+d/2\right)}{\frac{1}{2}\left(\ln\left|{\Sigma_{Y}}_{n}^{s}\right|-\ln\left|\Sigma_{Y_{n}^{s}|X_{n}^{s}}\right|\right)}\right)$$

We obtained the formula for the drift term, $\nabla_{Y}G\left(X_{n}^{s},Y_{n}^{s}\right)$ in the previous subsection. There are a few more statistical quantities we need to calculate. We obtain them thus - In our Notch Delta Jagged systems (note that d=2),

$$X_{n}^{s}=\left(\begin{array}{c} N\\ L \end{array}\right)\;\text{and}\;Y_{n}^{s}=\left(\begin{array}{c} N_{ext}\\ L_{ext} \end{array}\right)$$

##### Quadratic term correction in β calculation

There is initially an additional term of the form ${\left(Y_{n}^{s}-F\left(Y_{n}^{s}\right)\right)}^{T}\Sigma_{Y}^{-1}\left(Y_{n}^{s}-F\left(Y_{n}^{s}\right)\right)$ in the numerator of β that we assumed went to zero. We calculated it as a correction term - $F\left(Y_{n}^{s}\right)$ was estimated to be the average of a cells’ neighbours’ $Y_{n}^{s}$’s and the term hence calculated. We obtained values of the order $10^{-17}$ (indeed, comparable to machine precision). We can therefore conclude that the assumption to neglect this term is well grounded.

##### Calculating ${\Sigma_{Y}}_{n}^{s}$

This statistical term appears in the denominator as well as the numerator (as $S\left(Y_{n}^{s}\right)=\frac{1}{2}\ln\left({\left(2\pi e\right)}^{2}\left|{\Sigma_{Y}}_{n}^{s}\right|\right)$). By the above definition of the $Y_{n}^{s}$ variable, we have,

(Equation 41) $${\Sigma_{Y}}_{n}^{s}=\left(\begin{array}{cc} Cov\left(N_{ext},N_{ext}\right) & Cov\left(N_{ext},L_{ext}\right)\\ Cov\left(L_{ext},N_{ext}\right) & Cov\left(L_{ext},L_{ext}\right) \end{array}\right)$$

where for each entry of the matrix, the covariances of two variables are calculated over the whole lattice. $\left|{\Sigma_{Y}}_{n}^{s}\right|$ is simply the determinant of the above matrix.

##### Calculating $\Sigma_{Y_{n}^{s}|X_{n}^{s}}$

This is a conditional covariance matrix and its computation is slightly more involved. For multivariate normal distributions - which we have assumed throughout our treatment of all these variables, the conditional covariance matrix is given by the following identity -,

(Equation 42) $$\Sigma_{Y_{n}^{s}|X_{n}^{s}}=\Sigma_{Y_{n}^{s}}-\Sigma_{Y_{n}^{s}X_{n}^{s}}{\left(\Sigma_{X_{n}^{s}}\right)}^{-1}\Sigma_{X_{n}^{s}Y_{n}^{s}}$$

For our Notch Delta Jagged system,

(Equation 43) $$\Sigma_{X_{n}^{s}}=\left(\begin{array}{cc} Cov\left(N,N\right) & Cov\left(N,L\right)\\ Cov\left(L,N\right) & Cov\left(L,L\right) \end{array}\right)$$

(Equation 44) $$\Sigma_{X_{n}^{s}Y_{n}^{s}}=\left(\begin{array}{cc} Cov\left(N,N_{ext}\right) & Cov\left(N,L_{ext}\right)\\ Cov\left(L,N_{ext}\right) & Cov\left(L,L_{ext}\right) \end{array}\right)$$

(Equation 45) $$\Sigma_{Y_{n}^{s}X_{n}^{s}}=\left(\begin{array}{cc} Cov\left(N_{ext},N\right) & Cov\left(N_{ext},L\right)\\ Cov\left(L_{ext},N\right) & Cov\left(L_{ext},L\right) \end{array}\right)$$

Thus, we can find $\left|\Sigma_{Y_{n}^{s}|X_{n}^{s}}\right|$ by taking the determinant of the above conditional covariance matrix.

##### Calculating c

This is a trivial calculation - we know d=2 here and the characteristic levels of Notch and the ligands can be taken to be 5000 units/cell. Thus,

$$c=\ln\;M+d/2=\ln\left(5000\sqrt{1^{2}+1^{2}}\right)+2/2=≃7.071\times 10^{3}$$

Having thus obtained the methods for calculating each of the terms in our formula for β, we are now in a position to calculate β over sweeps of the Notch Delta Jagged landscape.

#### Phase space of other terms in β formula

In Figure S2, we present how the individual terms that contribute to the analytical β calculation are quantified in the $\left(D_{0},J_{0}\right)$ phase space.

#### Calculation details for β=0 condition

In the following, we detail the calculations for deriving the condition for β=0:

$$\ln\left|\frac{\nabla_{X}G\left(X_{n}^{s},Y_{n}^{s}\right)}{\nabla_{Y}G\left(X_{n}^{s},Y_{n}^{s}\right)}\right|<0\text{.}$$

##### Approximation of the internal variable variance

Consider a dynamical system where a cell with internal variables $X_{n}^{t}$ in a microenvironment with external variables $Y_{n}^{t}$ is subject to the following ODE,

(Equation 46) $$\dot{X_{n}^{t}}=G\left(X_{n}^{t},Y_{n}^{t}\right)+\zeta \left(t\right)$$

Taking Taylor expansion around the stationary point $\left(X_{n}^{s},Y_{n}^{s}\right)$, where the system is at equilibrium,(Equation 47)

Taking squares and integrating on both sides,

(Equation 48) $$\begin{array}{c} \int {\left(X_{n}^{t}-X_{n}^{s}\right)}^{2}P\left(X_{n}^{t},Y_{n}^{t}\right)dX_{n}^{t}dY_{n}^{t}={\left|\frac{\nabla_{Y}G\left(X_{n}^{s},Y_{n}^{s}\right)}{\nabla_{X}G\left(X_{n}^{s},Y_{n}^{s}\right)}\right|}^{2}\int {\left(Y_{n}^{t}-X_{n}^{s}\right)}^{2}P\left(X_{n}^{t},Y_{n}^{t}\right)dX_{n}^{t}dY_{n}^{t}\\ \begin{array}{c} \sigma_{X}^{2}={\left|\frac{\nabla_{Y}G\left(X_{n}^{s},Y_{n}^{s}\right)}{\nabla_{X}G\left(X_{n}^{s},Y_{n}^{s}\right)}\right|}^{2}\sigma_{Y}^{2}. \end{array} \end{array}$$

##### Upper bound for entropy, S(X) from dynamics

Now, we know the mean and variance of our cell’s internal variables, $X_{n}^{s}$ from Equations 47 and 48. We can therefore assume $P\left(X_{n}^{s}\right)$ to be the maximally entropic probability distribution for the given constraints, which is the normal distribution. i.e.

(Equation 49) $$\begin{array}{l} P\left(X_{n}^{s}\right)∼N\left(X_{n}^{s}-\frac{\nabla_{Y}G\left(X_{n}^{s},Y_{n}^{s}\right)}{\nabla_{X}G\left(X_{n}^{s},Y_{n}^{s}\right)}\left(Y_{n}^{t}-Y_{n}^{s}\right),{\left|\frac{\nabla_{Y}G\left(X_{n}^{s},Y_{n}^{s}\right)}{\nabla_{X}G\left(X_{n}^{s},Y_{n}^{s}\right)}\right|}^{2}\sigma_{Y}^{2}\right)\\ \Rightarrow S\left(X_{n}^{s}\right)\leq \frac{1}{2}\ln\left({\left(2\pi e\right)}^{d}\sigma_{X_{n}^{s}}^{2}\right)\\ \begin{array}{c} S\left(X_{n}^{s}\right)\leq \frac{1}{2}\ln\left({\left(2\pi e\right)}^{d}{\left|\frac{\nabla_{Y}G\left(X_{n}^{s},Y_{n}^{s}\right)}{\nabla_{X}G\left(X_{n}^{s},Y_{n}^{s}\right)}\right|}^{2}\sigma_{Y_{n}^{s}}^{2}\right) \end{array} \end{array}$$

where, d is the dimension of the variable $X_{n}^{t}$ and $\sigma_{Y_{n}^{s}}^{2}$ is the variance of $Y_{n}^{s}$. Here, we make the implicit assumption that $Y_{n}^{t}$ is also normally distributed. Thus, its variance, $\sigma_{Y_{n}^{s}}^{2}$, corresponds to that of a multivariate normal distribution. i.e. $\sigma_{Y_{n}^{s}}^{2}=\left|{\Sigma_{Y}}_{n}^{s}\right|$, the determinant of its covariance matrix.

##### Phase space boundary calculation

We know that the boundary between the positive and negative β regimes would be given by β=0. Thus, to theoretically ground the observation from Figure 7, we are required to show that,

$$\beta =0\Rightarrow \ln\left|\frac{\nabla_{X}G\left(X_{n}^{s},Y_{n}^{s}\right)}{\nabla_{Y}G\left(X_{n}^{s},Y_{n}^{s}\right)}\right|<0$$

At the phase boundary, we are given β=0. Recalling Equation 26,

(Equation 50) $$\begin{array}{l} \Rightarrow S\left(X_{n}^{s}\right)=\ln\;Z_{I}\leq S\left(Y_{n}^{s}\right)+\ln\left|\frac{\nabla_{Y}G\left(X_{n}^{s},Y_{n}^{s}\right)}{\nabla_{X}G\left(X_{n}^{s},Y_{n}^{s}\right)}\right|\\ \Rightarrow \ln\left|\frac{\nabla_{Y}G\left(X_{n}^{s},Y_{n}^{s}\right)}{\nabla_{X}G\left(X_{n}^{s},Y_{n}^{s}\right)}\right|\geq \ln\;Z_{I}-S\left(Y_{n}^{s}\right)\\ ∴\begin{array}{c} \ln\left|\frac{\nabla_{X}G\left(X_{n}^{s},Y_{n}^{s}\right)}{\nabla_{Y}G\left(X_{n}^{s},Y_{n}^{s}\right)}\right|\leq S\left(Y_{n}^{s}\right)-\ln\;Z_{I} \end{array} \end{array}$$

Forβ=0

$$\ln\;Z_{I}=\int e^{\beta I\left(Y_{n}^{s},X_{n}^{s}=x\right)}dX_{n}^{s}=\int dX_{n}^{s}=M$$

where M is some characteristic number of molecules $∼10^{3}$. Thus,

$$\ln\left|\frac{\nabla_{X}G\left(X_{n}^{s},Y_{n}^{s}\right)}{\nabla_{Y}G\left(X_{n}^{s},Y_{n}^{s}\right)}\right|\leq S\left(Y_{n}^{s}\right)-M$$

We can see from the phase portrait of $\log\left|\Sigma_{Y_{n}^{s}}\right|$ that,

(Equation 51) $$\log\left|\Sigma_{Y_{n}^{s}}\right|∼\left(-8,25\right)$$

(Equation 52) $$\Rightarrow \ln\left|\Sigma_{Y_{n}^{s}}\right|∼\left(\frac{-8}{2.303},\frac{25}{2.303}\right)$$

(Equation 53) $$\Rightarrow S\left(Y_{n}^{s}\right)=\frac{1}{2}\ln\left({\left(2\pi e\right)}^{2}\left|\Sigma_{Y_{n}^{s}}\right|\right)∼\left(1.10101,8.26558\right)$$

(Equation 54) $$∴\ln\left|\frac{\nabla_{X}G\left(X_{n}^{s},Y_{n}^{s}\right)}{\nabla_{Y}G\left(X_{n}^{s},Y_{n}^{s}\right)}\right|≦S\left(Y_{n}^{s}\right)-M\ll 0$$

Therefore,

(Equation 55) $$\beta =0\Rightarrow \ln\left|\frac{\nabla_{X}G\left(X_{n}^{s},Y_{n}^{s}\right)}{\nabla_{Y}G\left(X_{n}^{s},Y_{n}^{s}\right)}\right|<0$$

We could arrive at the phase boundary result theoretically/empirically as well.

#### Spatial patterning quantification via structure factor

To quantify the correspondence between population distributions (captured by the modality index) and spatial patterning, we follow strategy based on the spatial Fourier transform (FT). We first obtain the two dimensional FTs of the spatial pattern. These are plotted in Figure S3B.

$$I_{FFT}\left(k\right)=\sum_{x}\sum_{y}I_{NDJ}\left(x,y\right)e^{-2\pi i\left(k_{x}x+k_{y}y\right)}$$

We then obtain the amplitude spectrum - by averaging all the FT values for a given wavenumber $k=\left(k_{x},k_{y}\right)$. (All the lattice points at a given radial distance, equidistant from the origin of the Fourier transform lattice correspond to the same magnitude of |k|). The amplitude spectrum when squared gives us the power spectrum, shown in Figure S3A.

$$p_{\kappa }\left(k\right)=\sum_{x}\sum_{y}I_{FFT}{\left(k\right)}^{2}\delta \left(\sqrt{k_{x}^{2}+k_{y}^{2}}-k\right)$$

For a given pattern, we integrate the area under the curve of its power spectrum, $AUC_{NDJ}$ and “normalise” it by subtracting the area under the curve of a random reference spatial pattern $AUC_{\mathrm{ran}dom}$.

$$AUC_{NDJ}=\sum_{k}p_{\kappa }\left(k\right)$$

We see in Figure S3A that while Delta dominated chessboard patterns have a larger area under the curve (corresponding to β<0) and Jagged dominated patterns have smaller areas (corresponding to β>0), along the phase boundary (where β≈ 0, the $AUC_{NDJ}$ is almost equal to $AUC_{\mathrm{ran}dom}$. Thus, the phase diagram of this metric, $AUC_{NDJ}-AUC_{random}$ which captures the spatial patterning, has an almost one-to-one correspondence with the phase diagram of both, modality index and calculated β values (Figure 8).

#### Parameter values for Notch Delta Jagged and LEUP simulations

##### Notch Delta Jagged simulations

These are taken with reference to,^4^

1. Basal transcription rates:

$$\begin{array}{c} N_{0}=1400\\ D_{0}\in \left[500,2000\right]\\ J_{0}\in \left[200,1300\right] \end{array}$$

It is also mentioned that the largest number of Notch proteins as well as Jagged/Delta molecules in a cell are ∼5000. Thus,

$$M=\left|X_{n}^{s}\right|=5000\sqrt{2}\approx 7\times 10^{3}$$

2. Rate constants:

$$\gamma =0.1\;\gamma_{I}=0.5$$

$$k_{T}=5\times 10^{-5}\;k_{C}=5\times 10^{-4}$$

3. Shifted Hill function parameters:

$$\begin{array}{c} \text{Threshold}\;\text{value}\;\text{of}\;\text{NICD},I_{0}=200\\ \text{Co}-\text{operativity},n_{N}=2.0\;n_{D}=2.0\;n_{J}=5.0\\ \text{Fold}\;\text{change},\lambda_{N}=2.0\;\lambda_{J}=2.0\;\lambda_{D}=0.0 \end{array}$$

Note that a shifted Hill function is of the form,

$$H^{s}\left(I\right)=\frac{1+\lambda {\left(\frac{I}{I_{0}}\right)}^{n}}{1+{\left(\frac{I}{I_{0}}\right)}^{n}}$$

4. Lattice parameters:

$$\begin{array}{c} \text{Size}\;\text{of}\;\text{Lattice}=25\times 25\\ dt=0.01s\\ T=300s\\ \text{Number}\;\text{of}\;\text{iterations}=300/0.01=30000 \end{array}$$

LEUP simulations,

$$\begin{array}{c} \text{Size}\;\text{of}\;\text{Lattice}=25\times 25\\ dt=0.01s\\ T=50s\\ \text{Number}\;\text{of}\;\text{iterations}=50/0.01=5000 \end{array}$$

### Quantification and statistical analysis

No statistical tests were performed in the study.
