# Supplementary material for: Microenvironmental entropy dynamics analysis reveals novel insights into Notch-Delta-Jagged decision-making mechanism
Source: iScience. 2024 Aug 21;27(9):110569. doi: 10.1016/j.isci.2024.110569 (PMC11420447; doi:10.1016/j.isci.2024.110569)
Supplement: Document S1. Figures S1–S3 and Table S1 [file mmc1.pdf]

iScience, Volume *27*

Supplemental information

Microenvironmental entropy dynamics analysis reveals novel insights into Notch-Delta-Jagged decision-making mechanism

Aditi Ajith Pujar, Arnab Barua, Partha Sarathi Dey, Divyoj Singh, Ushasi Roy, Mohit Kumar Jolly, and Haralampos Hatzikirou

S1: Supplamentary Information (S.I.)

Supplamentary Material

Table No. 1:

List of Symbols	
**Symbol**	**Explanation**
$\mathbf{Y}_n^t$	Microenvironment variables of n-th cell at time t
$\mathbf{Y}_n^s$	Microenvironment variables of n-th cell in steady state
$\mathbf{X}_n^t$	Internal variables of n-th cell at time t
$\mathbf{X}_n^s$	Internal variables of n-th cell in steady state
r	Radius of sensitivity
β_n	Sensitivity of n-th cell
N_n^t	Amount of Notch molecules in n-th cell at time t
D_n^t	Amount of Delta molecules in n-th cell at time t
J_n^t	Amount of Jagged molecules in n-th cell at time t
I_n^t	Amount of NICD complex molecules in n-th cell at time t
N_{ext}	Amount of Notch molecules in the neighbourhood of n-th cell
D_{ext}	Amount of Delta molecules in the neighbourhood of n-th cell
J_{ext}	Amount of Jagged molecules in the neighbourhood of n-th cell

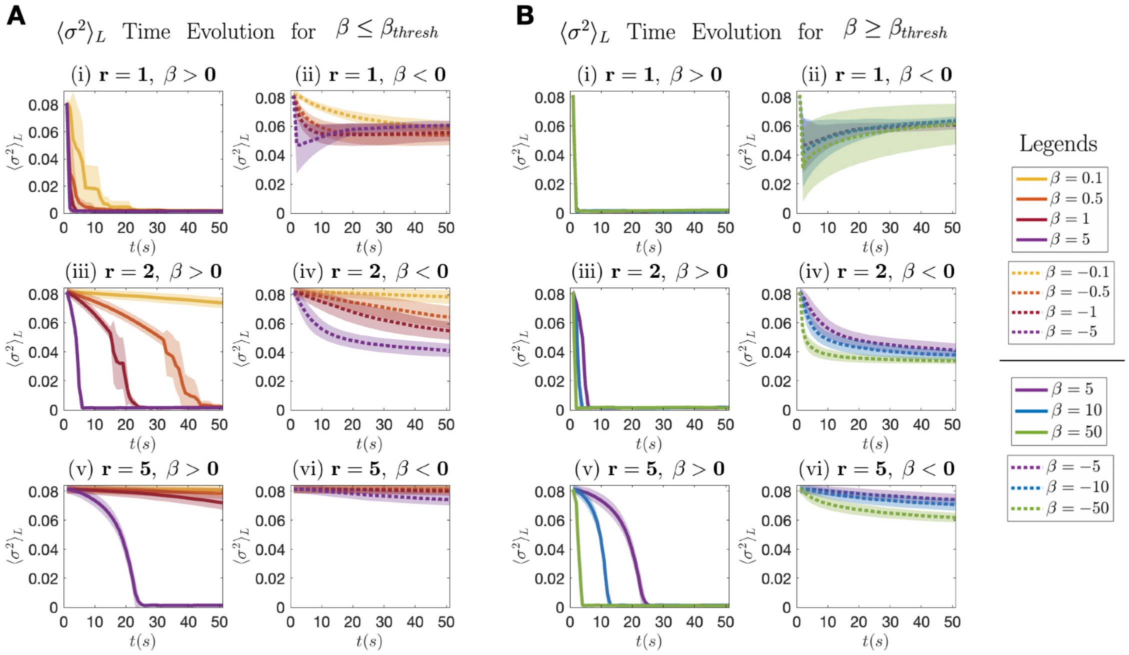

Figure S1: The study of average micronevironmental uncertainty over time in different sensing radius and sensitivity. The shading corresponds to the standard deviation when averaged over 5 simulations. We take two sets of β, *(A)* $|\beta| \leq |\beta_{thresh}|$ where we see that $\langle\sigma^2\rangle_L$ not changing much over time corresponds to cells taking random decisions, having not begun sensing yet. We see that the threshold sensitivity increases as r increases (due to signal attenuation). Secondly, for $r = 1$, $\langle\sigma^2\rangle_L$ increases (decreases) for $\beta < 0$ ($\beta > 0$) systems. We also note that for *(B)* $|\beta| \geq |\beta_{thresh}|$, most systems behave monotonically. As $|\beta|$ increases, they merely reach their steady states sooner. However, $(r = 1, \beta < 0)$ systems show non-monotonic behaviours. For them, $\langle\sigma^2\rangle_L$ settles to a lower steady state as $|\beta|$ increases due to the emergence of trimodality. Related to Fig. 4

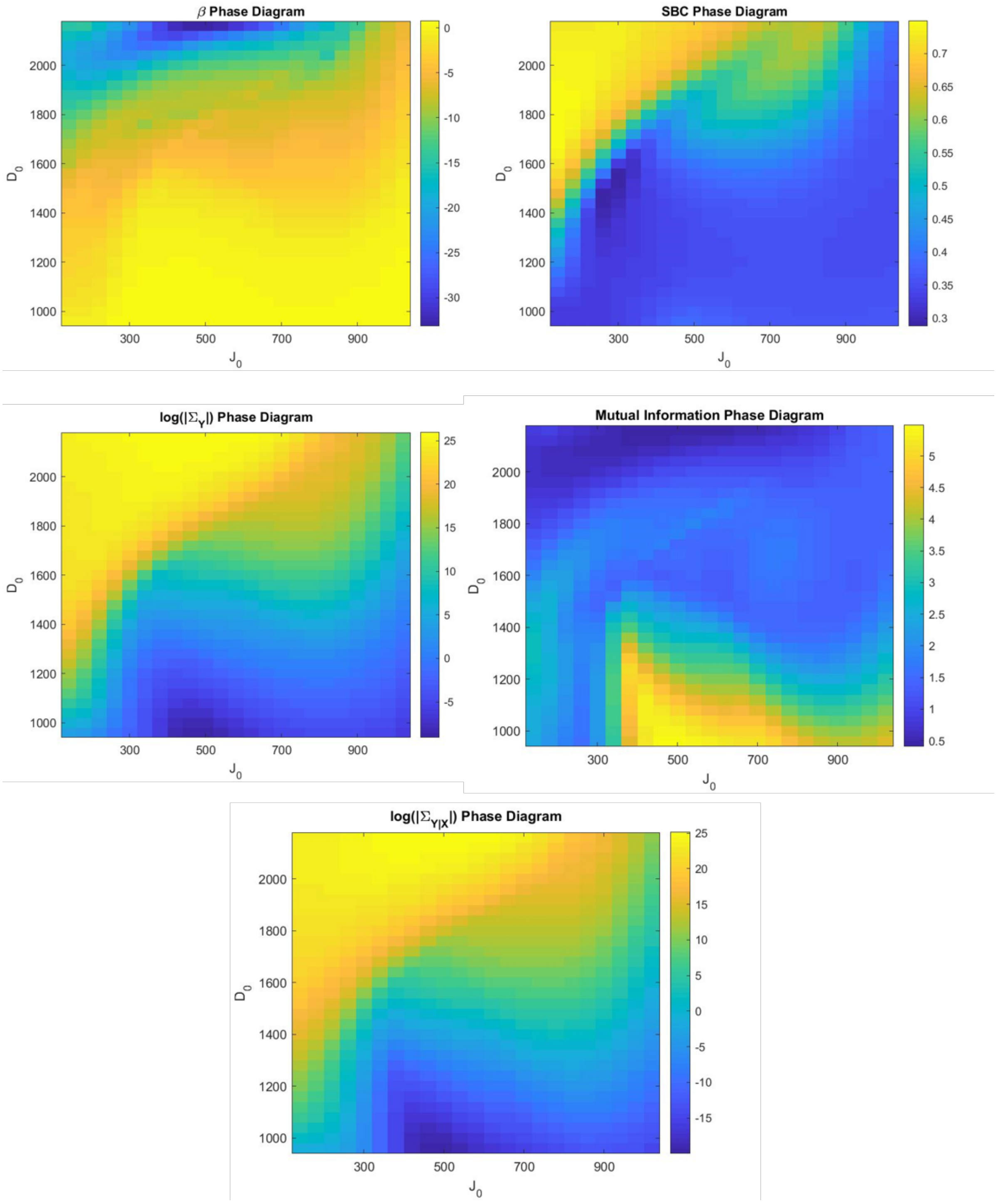

Figure S2: Phase diagrams of other relevant quantities. Here we plot the phase diagram in the (D_0, J_0) phase space with respect to $log(\mid \Sigma_Y \mid)$, mutual information and $log(\mid \Sigma_{Y|X} \mid)$. Related to Fig. 6

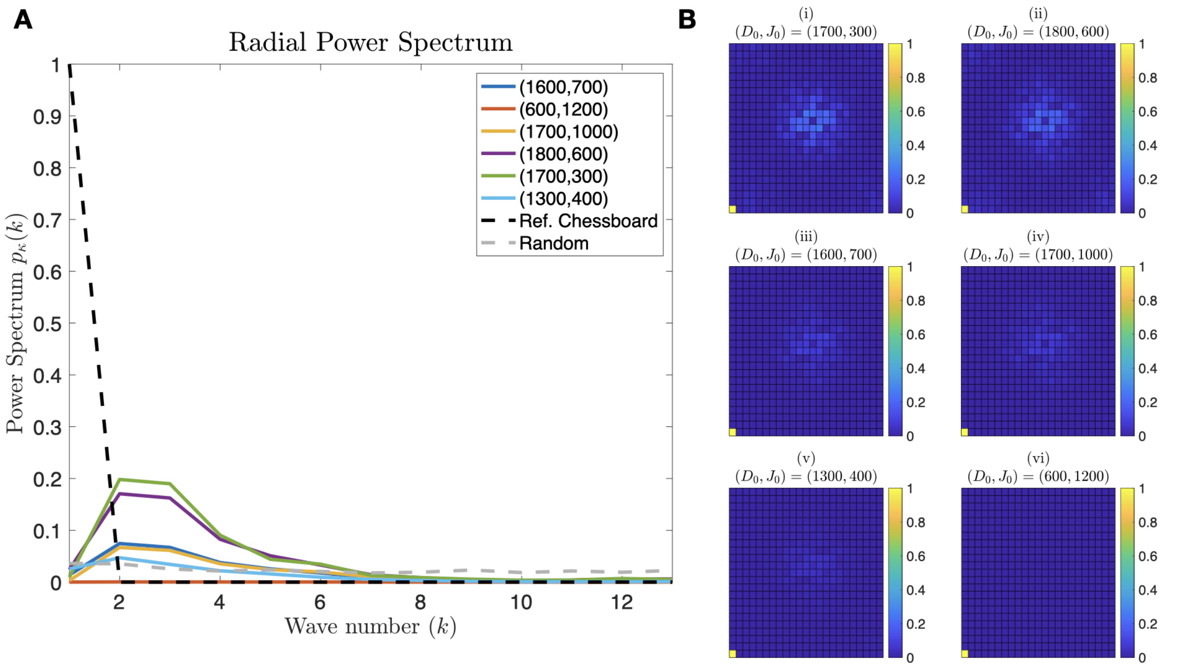

Figure S3: The plot of power spectra in different (D_0, J_0) space. *(A)* Power spectra of different spatial patterns emerging in Notch Delta Jagged signalling. *(B)* The normalised 2D FFTs of NICD levels various Notch Delta Jagged patterns. The power spectra are calculated over this. Related to Fig. 8
